# Excess glutamine rewires endothelial cell metabolism

**DOI:** 10.1007/s11306-026-02515-4

**Published:** 2026-08-22

**Authors:** Marzyeh Kheradmand, Xinyao Zhou, Funke Okunrinboye, Ganesh Sriram, Alisa Morss Clyne

**Affiliations:** 1 Fischell Department of Bioengineering, University of Maryland, 4224 A. James Clark Hall, 8278 Paint Branch Drive, College Park, MD 20742 USA; 2 Chemical and Biomolecular Engineering, University of Maryland, College Park, MD 20742 USA

## Abstract

**Introduction:**

Glutamine, the most abundant amino acid in the body, is a key metabolic substrate for endothelial cells. Glutamine supplementation protects against cardiovascular disease in animal models and in humans; however, glutamine *in vitro* has inconsistent effects on endothelial function. Furthermore, little is known about how altered metabolite concentrations, for example excess glucose in hyperglycemia or excess glutamine in cell culture media, affect endothelial cell metabolism.

**Objectives:**

The objective of this study was to determine how physiological and supplemented glutamine affect endothelial metabolism in normal and high glucose conditions.

**Methods:**

Primary human coronary artery endothelial cells were cultured in varied glutamine concentrations and in normal and high glucose. Glutamine uptake and glutamate secretion were measured using a YSI bioanalyzer; oxidative respiration was assessed using a Seahorse Metabolic Analyzer; and glutamine carbon incorporation into the TCA cycle, amino acids, antioxidants, and other pathways was evaluated via liquid chromatography-mass spectrometry.

**Results:**

As extracellular glutamine increased, endothelial cells took up more glutamine, but glutamate secretion saturated above 2 mM glutamine. Excess glutamine was primarily stored intracellularly, although increasing extracellular glutamine concentration did increase oxidative respiration and TCA cycle isotope enrichment. We also observed increased glutamine incorporation into glutathione, UDP-GlcNAc, and amino acids. When total metabolite abundance was examined, intracellular succinate, unsaturated fatty acids, and one-carbon metabolism-related metabolites decreased with increasing glutamine.

**Conclusion:**

These findings demonstrate that excess extracellular glutamine reprograms endothelial metabolism, suggesting that glutamine supplementation should be used with caution in cardiovascular therapies and endothelial cell culture.

**Supplementary Information:**

The online version contains supplementary material available at https://doi.org/10.1007/s11306-026-02515-4.

## Introduction

Endothelial cell dysfunction is a hallmark of cardiovascular disease (CVD), the leading cause of mortality among subjects with diabetes (Kannel 1979; Haffner et al. 1998; Dubsky et al. 2023; Yang et al. 2024a). Endothelial dysfunction is tightly linked to metabolic activity (De Vriese et al., 2000; Marfella et al., 2001; Rohlenova et al., 2018; Citrin et al., 2025). While endothelial cells rely on glycolysis for up to 85% of their ATP production during normal function, excess glucose metabolism can lead to dysfunction by increasing oxidative stress, permeability, and inflammation (Nishikawa et al., 2000; Du et al., 2001; Hoshiyama et al., 2004; Clyne, 2021; Xiao et al., 2021). However, the way in which excess glucose metabolism in conditions such as diabetic hyperglycemia interacts with other metabolic pathways is not fully understood.

Glutamine is the most abundant amino acid in the body, and in healthy endothelial cells, glutamine metabolism is crucial for biomass production during vascular sprouting and for maintaining redox homeostasis (Mirveis et al., 2023). Endothelial cells import glutamine through the Na⁺-dependent antiporter SLC1A5 (ASCT2), which exchanges extracellular glutamine for an intracellular neutral amino acid, as well as the Na⁺-dependent symporters SLC38A1 and SLC38A2 (Liu et al., 2018), which use the inward sodium electrochemical gradient to bring glutamine into the cell (Mann et al., 2003; Bröer & Gauthier-Coles, 2022).

Once glutamine enters the endothelial cell, glutaminase 1 (GLS-1) hydrolyzes glutamine into glutamate and ammonia (Wu et al., 2000; Huang et al., 2017; Zheng et al., 2026). Glutamate is further metabolized into α-ketoglutarate (α-KG), either through glutamate dehydrogenase (GLUD1) or via transamination reactions. α-KG then enters the tricarboxylic acid (TCA) cycle for ATP and macromolecule production (Hinca et al., 2021). Glutamate derived from glutamine contributes to the formation of other amino acids, including asparagine via asparagine synthetase and proline from pyrroline-5-carboxylate (P5C) via P5C reductase (Wu et al., 2000; Li et al., 2022). Glutamine-derived glutamate is also essential to the endothelial cell antioxidant system via glutathione (GSH) synthesis. Glutamate, through the enzyme glutamate–cysteine ligase (GCL), conjugates with cysteine to form the dipeptide γ-glutamylcysteine. Glutathione synthetase (GS) then adds glycine to γ-glutamylcysteine, resulting in the final tripeptide, γ-L-glutamyl-L-cysteinylglycine, known as GSH (Lu, 2013). Thus, glutamine could impact endothelial function via central carbon metabolism and biosynthetic pathway and by boosting antioxidant defense.

Glutamine also interacts with other metabolic pathways such as the hexosamine biosynthetic pathway (HBP). The HBP integrates glucose and glutamine metabolism to generate UDP-N-acetylglucosamine (UDP-GlcNAc), a substrate required for protein O-GlcNAcylation and cell signaling regulation (Wellen et al., 2010; Paneque et al., 2023). Glutamine directly impacts UDP-GlcNAc production by providing the essential amide group required for glutamine: fructose-6-phosphate aminotransferase (GFAT) activity. In addition, glutamine provides carbon substrates necessary for UDP-GlcNAc synthesis, including acetyl-CoA and UTP (Darley-Usmar et al., 2012; Yang et al., 2026). UDP-GlcNAc influences cell signaling through post-translational protein modification via GlcNAcylation, which often occurs at the same serine and threonine residues targeted by phosphorylation (Sessa et al., 1990; Aulak et al., 2020; Basehore et al., 2021).

Finally, glutamine contributes to the ornithine cycle and one carbon (1 C) metabolism. Glutamine‑derived glutamate can be converted to ornithine via pyrroline‑5‑carboxylate synthase. When this ornithine is metabolized, endothelial cells synthesize polyamines, which are essential for cell proliferation, as well as proline, which is essential for collagen synthesis and vascular extracellular matrix integrity (Wu et al., 2000; Sivashanmugam et al. 2017; Li et al., 2026). Glutamine metabolism in the TCA cycle supplies precursors for serine and glycine, the main 1 C donors to 1 C metabolism (Ducker & Rabinowitz, 2017; Hwang et al., 2023). This glutamine‑supported 1 C flux sustains nucleotide synthesis, redox balance, and methylation capacity, thereby promoting endothelial cell proliferation, survival, and genetic regulation (Ducker & Rabinowitz, 2017; Petrova et al., 2023).

Glutamine supplementation may protect against CVD and related complications through several metabolic and anti-inflammatory mechanisms. In pulmonary artery endothelial cells, glutamine supplementation reduced hydrogen peroxide–induced injury, helping maintain cellular ATP and viability during high oxidative stress (Hinshaw & Burger, 1990). In animal models of endotoxin shock and severe injury, glutamine supplementation improved survival, enhanced immune function, and supported gut barrier integrity (Wischmeyer et al., 2001). However, glutamine shows inconsistent effects on endothelial function, with beneficial effects in some studies and detrimental effects in others (Hecker et al.; Meininger & Guoyao, 1990; Ellis et al., 2016; Kheradmand et al., 2026).

Although glutamine supplementation is used clinically to enhance vascular health, the endothelial cell metabolic adaptations in response to altered glutamine are not fully understood. In this study, we therefore examined how physiological and supplemented glutamine affect endothelial metabolism. We hypothesized that increasing glutamine concentration will increase TCA cycle flux in the forward and reverse directions, as well as amino acid synthesis and HBP flux. We supplemented endothelial cell culture media with 0–10 mM glutamine and measured changes in glutamine and glutamate levels. We then used a Seahorse Mito Stress Test to measure oxidative respiration and heavy isotope-labeled glutamine to track glutamine-derived metabolites by liquid chromatography-mass spectrometry. Understanding how glutamine systematically impacts endothelial cell metabolism may help identify novel metabolic targets for cardiovascular disease prevention.

## Materials and methods

### Endothelial cell culture

Primary human coronary artery endothelial cells (HCAEC, passage 5–9) were purchased from Lifeline Cell Technology. HCAECs were cultured in Endothelial Growth Medium-2 (EGM-2; Lonza) supplemented with 1% penicillin-streptomycin (PS; Thermo Fisher Scientific, 15140163), 10% fetal bovine serum (FBS; Cytiva, SH30088) and 1% glutamine (Fisher Scientific, 25–030-081). Cells were passaged into appropriate well plates and incubated in EGM-2 media until confluent. Cells were then switched to Dulbecco’s Modified Eagle Medium (DMEM) without phenol red, glucose, or glutamine (Gibco, A1443001) supplemented with Endothelial SingleQuots (Lonza, CC-4176), 5.5 or 15 mM D-glucose (Sigma-Aldrich, G8270), and 0–10 mM glutamine for 24 h. Low glutamine concentrations (0.5–2 mM) were used to simulate *in vivo* circulating glutamine, while higher glutamine concentrations were used to replicate the elevated glutamine in some *in vitro* cell culture media. L-glucose or mannitol were used as osmotic controls to match the osmolarity of the corresponding glutamine and glucose additions.

### Glutamine uptake and glutamate secretion

A YSI 2950 Biochemistry Analyzer (Yellow Springs Instruments, 527690) was used to measure media glucose, lactate, glutamine, and glutamate concentration. At 0 and 24 h of glutamine treatment, 150 µL media was collected and placed in a 96 well-plate (CELLTREAT; 229196) for analysis by YSI. Glucose and glutamine uptake and lactate and glutamate secretion were calculated as the difference between the 0 and 24 h concentrations.

### Glutaminase analysis

A Western blot was used to detect changes in HCAEC glutaminase. After glutamine treatment, cells were washed with phosphate-buffered saline (PBS; Thermo Fisher Scientific; 70011069) and lysed with RIPA buffer (Thermo Fisher Scientific; 89901) containing Halt protease and phosphatase inhibitor cocktail (Thermo Fisher Scientific; 78440) and ethylenediaminetetraacetic acid (EDTA; Sigma-Aldrich; E9884). The cell lysate was centrifuged for 15 min at 17,000 × g at 4 °C to remove cellular debris. The supernatant was collected, and the protein concentration was quantified using a BCA assay (Thermo Fisher Scientific; 23225). 3.5 µg protein was loaded into each well of a NuPAGE 4%−12% Bis-Tris gel (Thermo Fisher Scientific; NP0323BOX) for separation by SDS-PAGE. Proteins were then transferred to a polyvinylidene difluoride membrane (Thermo Fisher Scientific; IB34001 × 3) using an iBlot 3 (Thermo Fisher Scientific). Membranes were blocked in 5% bovine serum albumin (BSA; Sigma-Aldrich; A7906) in tris buffered saline (TBS; Fisher Scientific; BP24711) with 0.5% Tween (TBS-T; Thermo Fisher Scientific; 85115) for 1 h at room temperature. Blots were then incubated with primary antibodies in 1% BSA in TBS-T overnight at 4 °C. Primary antibodies included glutaminase-1 (1:1000; Abcam; EP7212) and β-actin (1:1000; Santa Cruz Biotechnology; sc-47778). The next day, membranes were washed with TBS-T and incubated with the appropriate anti-rabbit (Promega; W4011) or anti-mouse (Promega; W4021) secondary antibodies (1:2000) for 2 h. Protein bands were visualized by SuperSignal West Pico PLUS Chemiluminescent Substrate kit (Thermo Fisher Scientific; 34578) and imaged using an Alpha Innotech Fluorchem Imager. AlphaView SA 3.4.0 was used to quantify band intensity.

### Oxidative respiration

HCAEC oxidative respiration was measured using a Seahorse Mito Stress Test (Agilent; 103015). HCAEC were seeded at 50,000 cells/well in Seahorse XF96 cell culture plates and incubated overnight to allow cells to adhere to the plate. The next day, DMEM with 0, 0.5, or 2 mM glutamine and 5.5 or 15 mM D-glucose or the appropriate osmotic controls was added to the wells. After 24 h, the assay was performed on a Seahorse XFe96 (Agilent) according to the manufacturer’s protocols. Wave software (Agilent) was used to calculate basal respiration, maximal respiration, spare capacity, and non-mitochondrial oxygen consumption from the measured oxygen consumption rate (OCR). Following the assay, cells were stained with DAPI for 10 min, and cell nuclei were counted. Seahorse values were normalized to cell count for each well. Experiments performed on different days were normalized to the average OCR of the 0 mM glutamine condition.

### Isotope tracing via liquid chromatography-tandem mass spectrometry (LC-MS/MS)

To gain detailed insights into intracellular glutamine metabolism, we designed a parallel isotope-labeling experiment with 1-^13^ C and 5-^13^ C glutamine. 1-^13^C-glutamine was used to trace glutamine entry into the reductive (reverse) carboxylation pathway, as the C1 label is released as CO₂ during forward TCA cycle flux. 5-^13^C-glutamine was used to identify forward TCA–derived metabolites. 0, 1, 2, or 5 mM 1-^13^C-glutamine (CLM-3612, Cambridge Isotope Laboratory) or 5-^13^ C-glutamine (CLM-1166, Cambridge Isotope Laboratory) were added to confluent HCAECs in 6 well-plates in supplemented DMEM (5.5 mM glucose) for 24 h. Endothelial cells were previously determined to reach isotopic steady state by this time(Moiz et al., 2021). Metabolites were then extracted in 500 µL ice-cold 80:20 methanol: water at −80 °C for 15 min. Cell lysates were scraped in the extraction solvent, transferred to 1.7 mL micro centrifuge tubes, and centrifuged at 17,000 × g for 15 min at 4 °C to pellet cell proteins. Finally, the pellet was lysed in 50 µL RIPA buffer, and protein concentration was measured by BCA for normalization. The supernatant was collected, stored at −80 °C, and analyzed by LC-MS/MS in the University of Colorado School of Medicine Metabolomics Core. Metabolomics analysis was performed using a Vanquish UHPLC system (Thermo Fisher Scientific) coupled to an Orbitrap Exploris 120 mass spectrometer (Thermo Fisher Scientific). Samples (10 µL) were injected onto a 2.1 × 100 mm, 1.7 μm particle size Waters Acquity BEH C18 column at 45 °C and separated using a 5-minute reversed-phase gradient, based on a previously described method (Nemkov et al., 2019). For negative ion mode, mobile phase A consisted of water with 10 mM ammonium acetate (NH_4_OAc), and mobile phase B was a 1:1 mixture of methanol and acetonitrile with 10 mM NH_4_OAc. For positive ion mode, mobile phase A was water with 0.1% formic acid, while mobile phase B was acetonitrile with 0.1% formic acid.

In both ionization modes, the UHPLC separation followed the same gradient: 100% A (0.00 to 0.50 min); increased to 100% B (0.50 to 1.10 min); held at 100% B (1.10 to 2.75 min); returned to 100% A (2.75 to 3.00 min); and held at 100% A (3.00 to 5.00 min) for re-equilibration. The flow rate was maintained at 0.45 mL/min throughout the analysis. The mass spectrometer was run independently in both negative and positive ion modes, acquiring full MS scans over an *m/z* range of 65–975 at a resolution of 120,000. Source conditions included a sheath gas flow of 50 arbitrary units (Arb), auxiliary gas flow of 10 Arb, and spray voltages of 3 kV (negative mode) and 3.4 kV (positive mode). The instrument was calibrated prior to analysis using the Easy-IC internal standard (Thermo Fisher Scientific).

Sample run order was randomized. Technical replicates were injected throughout each sequence to assess analytical reproducibility and quality control. Raw files were converted to.mzXML format using RawConverter and analyzed with El-Maven (Elucidata) using the KEGG database for metabolite identification and peak integration, as previously described (Nemkov et al., 2017).

### Mass spectrometry data analysis

Natural abundance correction, multivariate analysis, and univariate analysis were performed in RStudio. The IsoCorrector 1.22.0 package was first used to correct the isotopologue natural abundance for each metabolite from the LC–MS/MS raw data. As we used one carbon labeled glutamine, labeling was expected to result primarily in M + 1 isotopologues. Consistent with this, higher order isotopologues (M + 2 and above) were not detected for most metabolites. Under these conditions, natural abundance correction using IsoCorrectoR may overcorrect the data. Therefore, both raw and natural abundance–corrected mass isotopologue distributions were analyzed. As both approaches showed consistent trends, raw mass isotopologue distributions are included in the supplementary data (Online resources 8 and 9), and the corrected mass isotopologue distributions are presented in the main figures. After correction, the labeled fraction was calculated by dividing the signal from labeled metabolites by the total signal (labeled + unlabeled). The mixOmics 6.28.0 package was then used for unsupervised multivariate analysis principal component analysis (PCA) as an exploratory tool. PCA score and loading plots were generated using the ggplot2 package in RStudio, and stackplots and barplots were created using GraphPad Prism 10.

### Media succinate

Media was collected from HCAEC cultured in 5.5 mM D-glucose DMEM with 0 or 5 mM glutamine for 24 h. Media was centrifuged for 10 min at 17,000 × g at 4 °C to remove cell debris. 200 µL supernatant was added to 800 µL ice cold 100% HPLC grade methanol, vortexed, and centrifuged at 17,000 × g at 4 °C for 15 min. The supernatant was transferred to a new tube and dried overnight using a CentriVap Vacuum Concentrator (Labconco). Samples were resuspended in 100 µL 80% methanol (Sigma-Aldrich, G6025-11VL) and transferred to mass spectrometry vials (Waters, 600000670). Extracellular succinate was analyzed using a Bruker maXis-II QTOF mass spectrometer (Bruker Daltonics) coupled to a Waters ACQUITY UPLC system. Chromatographic separation was performed on an Atlantis BEH C18 AX column (2.1 × 100 mm, 1.7 μm; Waters) maintained at 30 °C, with the sample manager held at 6 °C. A 5 µL injection volume was used. The mobile phases consisted of solvent A: water containing 0.1% formic acid and solvent B: acetonitrile containing 0.1% formic acid. The flow rate was 0.25 mL/min. The gradient program was: 0–2 min, 2% A; 2–11 min, linear increase to 90% A; 11–16 min, hold at 90% A; 16–17 min, return to 2% A; 17–20 min, re-equilibration at 2% A. Mass spectrometric detection was performed in negative electrospray ionization (ESI−) mode with a capillary voltage of 4,500 V. Data were acquired in full scan mode over an m/z range of 80–400 without MS/MS fragmentation. A 10 µM sodium succinate dibasic hexahydrate standard (Sigma-Aldrich; S2378) was injected at the beginning of each sequence to confirm both mass and retention time prior to sample analysis. Succinate was identified based on accurate mass and retention time relative to the standard. Extracted ion chromatograms were generated at m/z 116.9967 ± 0.02 Da, and the succinate peak eluted at 1.4 min.

### Succinate dehydrogenase activity assay

Succinate dehydrogenase (SDH) activity was measured using a commercially available SDH Activity Assay Kit (Abcam; ab228560) according to the manufacturer’s instructions. HCAECs were seeded at equal density in 100 mm culture dishes and grown to confluency in EGM-2 prior to each experiment. Cells were rinsed with PBS and then switched to DMEM supplemented with 1% PS, 10% FBS, 5.5 mM glucose, and either 0 or 5 mM glutamine for 24 h. Following treatment, cells were detached using trypsin, washed with cold PBS, and resuspended in 100 µL SDH assay buffer supplemented with 1% protease inhibitor cocktail and EDTA. Cells were lysed using a D1000 homogenizer (Benchmark Scientific), incubated on ice for 10 min, and then centrifuged at 10,000 × g for 5 min at 4 °C. The supernatant was collected for analysis. Protein concentration was determined using a BCA assay. 75 µg protein for each sample was loaded into a 96-well plate and adjusted to a final volume of 50 µL. The standard curve and reaction mixture were prepared according to the manufacturer’s protocol and added to the designated wells. Absorbance was measured at 600 nm in kinetic mode for 60 min at 2 min intervals using a Spark multimode plate reader (TECAN). SDH activity was calculated according to the manufacturer’s instructions using the linear portion of the kinetic curve.

### Statistical analysis

All statistical analyses were performed using GraphPad Prism 10. Data are presented as mean ± standard deviation (SD) unless otherwise indicated. For experiments involving multiple glutamine concentrations, statistical significance was determined using an ordinary one-way ANOVA. When all samples were compared to each other, a Tukey’s multiple comparisons test was used. When comparisons were made to a single control group (e.g., 0 mM glutamine), a Dunnett’s multiple comparisons test was used. For comparisons between two groups, a non-parametric Mann Whitney test was used. Statistical significance was defined as *p* < 0.05. The number of biological replicates (n) and the specific statistical test for each dataset are indicated in the corresponding figure legend.

## Results

### Glutamine uptake and glutamate secretion increased with glutamine concentration up to 2 mM glutamine

**Fig. 1:**
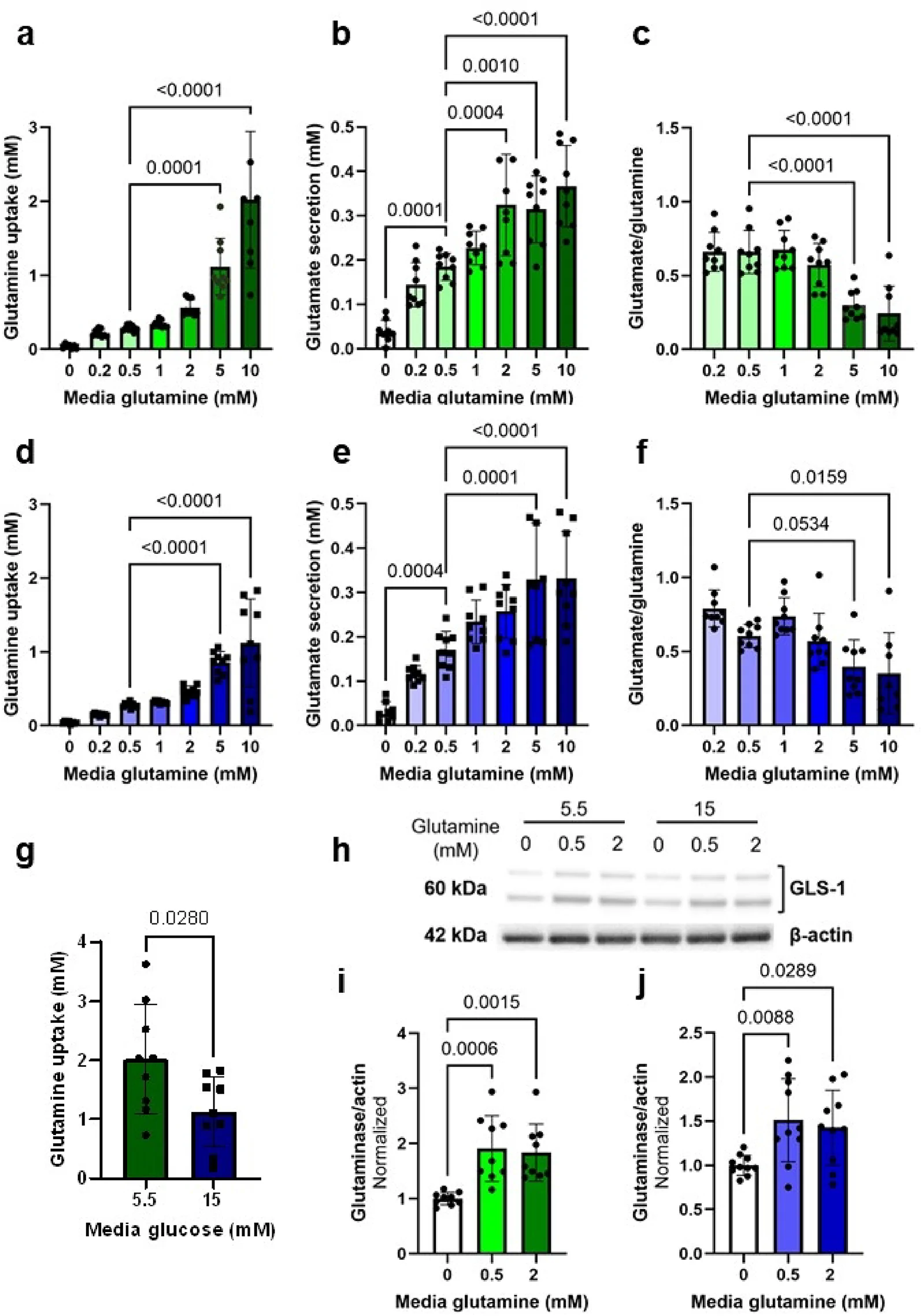
Glutamine uptake and glutamate secretion increased with glutamine concentration; however, glutamate secretion plateaued above 2 mM glutamine. HCAEC were incubated with 0–10 mM glutamine in normal (5.5) and high glucose (15 mM) for 24 h. Glutamine uptake and glutamate secretion were measured in normal (**a**, **b**) and high (**d**, **e**) glucose using a YSI bioanalyzer. The glutamate: glutamine ratio was determined for each condition in (**c**) normal and (**f**) high glucose. **g** Comparison of glutamine uptake in normal vs. high glucose at 10 mM glutamine. *n* = 9 biological replicates. **h** Representative glutaminase-1 (GLS-1) and β-actin Western blots, with quantification of (**i**) normal glucose and (**j**) high glucose. *n* = 9 biological replicates. Data were analyzed using an ordinary one-way ANOVA with a Dunnett post-hoc multiple comparisons test

We first determined how HCAECs take up glutamine in both 5.5 mM (normal) and 15 mM (high) glucose by adding 0–10 mM glutamine to the cell culture media. We used low glutamine concentrations to replicate physiological circulating levels and high glutamine concentrations to simulate *in vitro* cell culture media. HCAECs took up significantly more glutamine as media glutamine concentration increased in both normal and high glucose, as analyzed by one-way ANOVA (*p* < 0.0001; Fig. 1a, d). In normal glucose, HCAECs took up 5 and 10 times more glutamine, respectively, at 5 and 10 mM glutamine than at the physiological level of 0.5 mM glutamine (*p* < 0.0001; Fig. 1a). Similarly, in high glucose, HCAEC took up more than 4 times more glutamine in 5 and 10 mM glutamine as compared to 0.5 mM glutamine (*p* < 0.001; Fig. 1d). Glutamine uptake was not impacted by osmotic effects (Online resource 3). Glutamine uptake was only statistically different between normal and high glucose at 10 mM glutamine, where glutamine uptake in high glucose was around half the uptake in normal glucose (*p* = 0.0280; Fig. 1g).

We then measured secretion of the primary glutamine-derived metabolite, glutamate, into the cell culture media. Glutamate secretion also increased with increasing media glutamine concentration in both normal and high glucose conditions (*p* < 0.0001 by ANOVA; Fig. 1b, e). In normal glucose, glutamate secretion was about 10 times higher for HCAEC in 0.5 mM as compared to 0 mM glutamine (*p* = 0.0001) and about 2 times higher for HCAEC in 2, 5, or 10 mM glutamine as compared to 0.5 mM glutamine (*p* = 0.0004, 0.0010, and < 0.0001, respectively). In both normal and high glucose, HCAEC glutamate secretion did not change above 2 mM glutamine. Glutamate secretion was not impacted by osmotic effects (Online resource 3). There were no statistically significant differences in glutamate secretion for HCAEC in normal and high glucose at any glutamine concentration.

We calculated the ratio of glutamate secreted to glutamine uptake as a measure of glutaminolysis. From 0.2 to 2 mM glutamine, the glutamate: glutamine ratio remained statistically similar at around 0.7 for HCAEC in normal and high glucose. At 5 and 10 mM glutamine, the glutamate: glutamine ratio decreased to around 0.2 for HCAEC in normal glucose (*p* < 0.0001) and around 0.3 for HCAEC in high glucose (*p* = 0.0534 at 5 mM and *p* = 0.0159 for 10 mM glutamine). There were no statistically significant differences in the glutamate: glutamine ratio for HCAEC in normal and high glucose.

Glutaminase 1 (GLS-1) catalyzes glutamine deamidation to glutamate. We therefore determined how extracellular glutamine concentration affected GLS-1 protein by Western blot (Fig. 1h, j). As we did not observe significant changes in glutamate secretion at glutamine concentrations higher than 2 mM, we measured glutaminase protein levels at 0, 0.5 and 2 mM glutamine. HCAEC in normal glucose and 0.5 or 2 mM glutamine had twice as much GLS-1 compared to HCAEC in 0 mM glutamine (*p* = 0.0006 and *p* = 0.0015, respectively; Fig. 1i). Similarly, HCAEC in high glucose and 0.5 or 2 mM glutamine had 50% more GLS-1 compared to HCAEC in 0 mM glutamine (*p* = 0.0088 and *p* = 0.0289, respectively; Fig. 1j). GLS-1 protein was not higher at 2 mM glutamine as compared to 0.5 mM glutamine for HCAEC in normal or high glucose. These data suggest that ECs take up more glutamine as extracellular glutamine concentration increases but reach a limit of glutamate secretion, possibly due to a lack of additional GLS-1.

Since endothelial cells depend on glycolysis, we assessed glucose uptake and lactate secretion in HCAECs in increasing extracellular glutamine. Glutamine supplementation did not significantly alter glucose uptake (Online resource 1a, c). Lactate secretion increased with extracellular glutamine for HCAEC in both normal and high glucose (*p* = 0.0007 and *p* < 0.0001 by ANOVA for normal and high glucose, respectively; Online resource 1b, d). Lactate secretion at 10 mM glutamine was about 25% higher for HCAEC in normal and high glucose as compared to HCAEC in 0.5 mM glutamine. These data show that extracellular glutamine did not significantly change glycolysis, suggesting that the two metabolic pathways remain distinct.

### Glutamine increased oxidative respiration and isotope enrichment through the forward TCA cycle with limited impact on reverse carboxylation

**Fig. 2:**
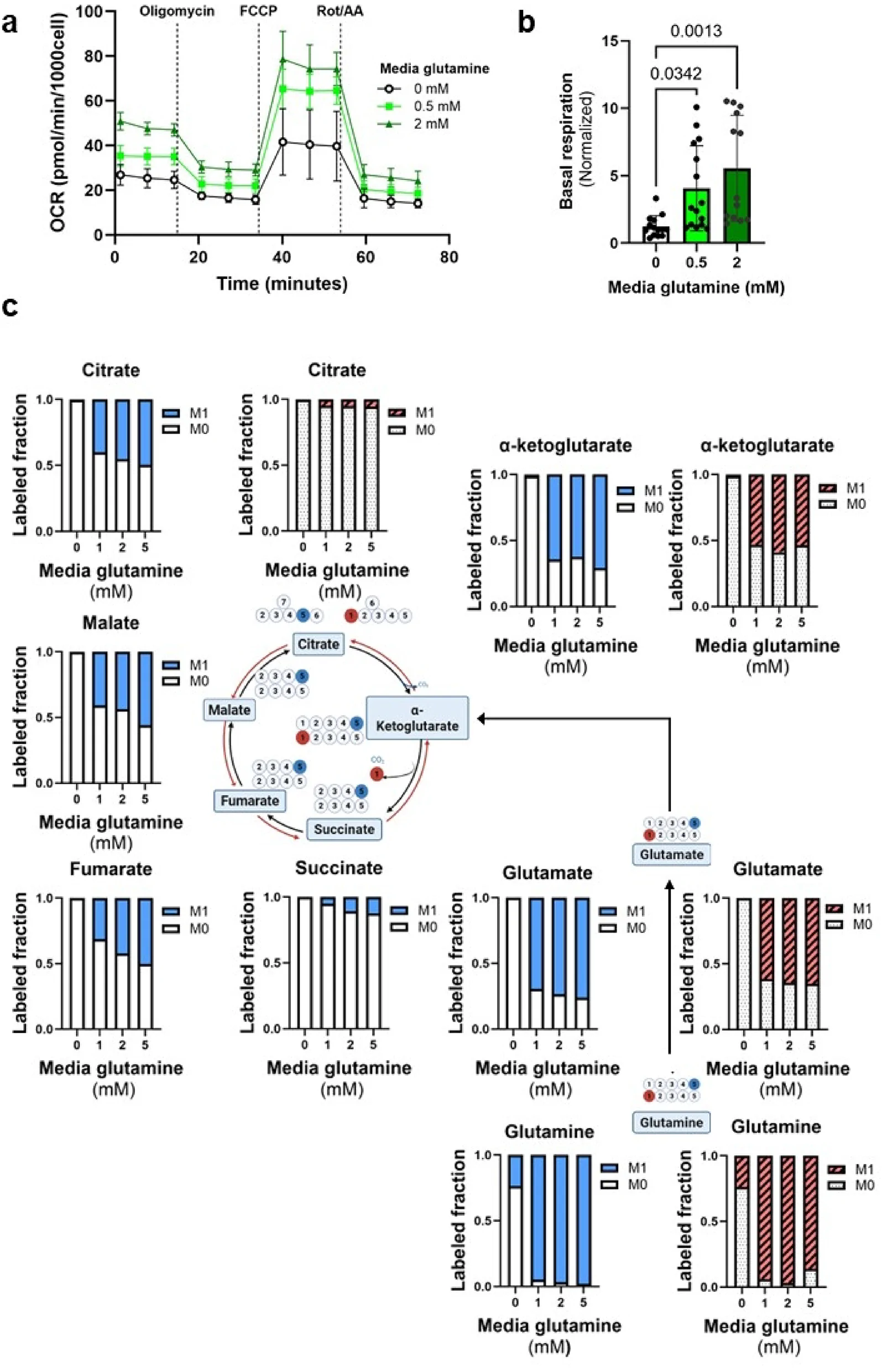
Glutamine increased endothelial oxidative respiration and isotope enrichment through the forward TCA cycle; reverse carboxylation also increased with glutamine but was limited overall. HCAECs were incubated for 24 h with 0, 0.5, or 2 mM glutamine in normal (5.5 mM) glucose culture. **a** Representative oxygen consumption rate (OCR) as measured by Seahorse Mito Stress test. **b** Basal respiration from three experiments, normalized to the 0 mM glutamine condition. *n* = 10–16 biological replicates. Data were analyzed using ordinary one-way ANOVA with a Tukey post-hoc multiple comparisons test. (c) HCAECs were cultured with 0, 1, 2, or 5 mM 1-^13^C- or 5-^13^ C-glutamine for 24 h, after which cells were collected and the isotope distribution was determined using LC-MS. Isotope labeled fractions for 5-^13^ C-glutamine metabolites are shown in blue. Isotope labeled fractions for 1-^13^ C-glutamine metabolites are shown in red with diagonal hatching. 5-^13^ C-glutamine was used to measure glutamine flux through the forward TCA cycle, since α-KG loses its first carbon as CO_2_ when it converts to succinate, thereby retaining the M1 label. 1-^13^ C-glutamine was used to measure reverse carboxylation, since when α-KG is converted to citrate in the reverse TCA cycle, the first labeled carbon is preserved. M0 and M1 refer to the number of ^13^C atoms incorporated into a metabolite, where M0 has no ^13^C and M1 has one ^13^C atom. *n* = 3 biological replicates. Created in BioRender. Kheradmand-Hajibashi, M. (2026) https://BioRender.com/gom6hqu

Glutamine is a primary carbon source for the endothelial TCA cycle. As we did not measure changes in glutamate secretion above 2 mM glutamine, we measured how 0, 0.5, or 2 mM extracellular glutamine impacted mitochondrial activity (Fig. 2a) using a Seahorse Mito Stress test. HCAEC oxygen consumption rate (OCR), a measure of mitochondrial oxidative respiration, increased with glutamine (*p* = 0.0016 for basal OCR and *p* < 0.0001 for all others by ANOVA). Basal OCR was five time higher for cells in 0.5 and 2 mM glutamine compared to 0 mM glutamine (*p* = 0.0342 and 0.0013, respectively; Fig. 2b). Maximal respiration, which measures mitochondrial ability to meet increased energy demand (Desler et al., 2012), and spare capacity, which is the difference between maximal and basal respiration, more than doubled for cells in 0.5 (*p* = 0.008) and 2 mM (*p* < 0.0001) glutamine relative to 0 mM glutamine (Online resource 2a, b). Similarly, cells in 0.5 and 2 mM glutamine had double the non-mitochondrial oxygen consumption of cells in 0 mM glutamine (*p* = 0.0003 and *p* < 0.0001, respectively; Online resource 2c), indicating increased cellular oxidase activity. Only spare respiratory capacity was statistically significantly higher for cells in 2 mM as compared to 0.5 mM glutamine (*p* = 0.0058). We observed a similar OCR response to glutamine in HCAECs cultured in high glucose (Online resource 2d-h). Osmolarity did not affect OCR for HCAECs in low glucose; however, osmolar effects increased OCR for HCAEC in high glucose (Online resource 4). Taken together, these data show that glutamine fuels endothelial mitochondrial activity, although the effect is limited at higher glutamine concentrations.

Since extracellular glutamine increased endothelial oxidative respiration, we used isotope-assisted tracing to get a more detailed understanding of how glutamine contributes to TCA cycle metabolites. We conducted all labeling experiments in normal glucose, as we observed no differences in TCA cycle activity for HCAEC in normal and high glucose. We used 1 mM glutamine instead of 0.5 mm glutamine to model physiological glutamine concentration due to concerns about our ability to detect isotope labeling at the lower concentration. We used 2 mM glutamine to model a commonly used endothelial cell culture media concentration and 5 mM glutamine to observe changes with additional glutamine. We used 1-^13^ C-glutamine and 5-^13^ C-glutamine parallel labeling to understand glutamine entry into forward and reductive carboxylation. In the forward (oxidative) TCA cycle, 1-^13^ C-glutamine loses its labeled C-1 carbon when α-KG is converted to succinate. However, in the reductive carboxylation pathway, the C-1 carbon from 1-^13^ C glutamine appears in citrate. Thus, we expect a significant M1 isotopomer abundance for citrate with no significant M1 isotopomer abundances for other TCA cycle metabolites in the 1-^13^ C glutamine experiment if reductive carboxylation is active. In contrast, 5-^13^ C-glutamine retains its labeled C-5 carbon in the forward TCA cycle. The C-5 carbon from 5- ^13^ C glutamine appears in C-1 or C-4 position of succinate (due to molecular symmetry). It then passes to the C-1 or C-4 position of fumarate, malate and oxaloacetate, finally ending up in the C-1 or C-6 position of citrate. In the next turn through the forward TCA cycle, the labeled carbon is lost as CO_2_. Therefore, we expect substantial M1 isotopomer abundances for all TCA cycle metabolites in the 5-^13^ C glutamine labeling experiment. Since the cells are likely to concurrently operate both the forward TCA cycle and reductive carboxylation, we expect to observe a superimposition of the labeling scenarios described above.

Our natural abundance corrected LC-MS isotope enrichment data (Fig. 2c) showed that nearly all intracellular glutamine was labeled in HCAEC treated with 1, 2, or 5 mM 1-^13^C-glutamine or 5-^13^ C-glutamine. 5-^13^ C-glutamate enrichment increased from 69% to 76%, and 5-^13^ C-α-KG enrichment increased from 64% to 71% with increasing extracellular glutamine. Labeled fraction of TCA metabolites in the forward TCA cycle also increased in a concentration dependent manner as extracellular glutamine increased from 1 to 5 mM, including succinate from 4% to 12%, fumarate from 31% to 50%, malate from 40% to 55%, and citrate from 39% to 49%. However, 1-^13^ C-citrate enrichment was less than 5% across all glutamine concentrations, indicating limited reductive carboxylation. Interestingly, succinate had the highest unlabeled pool among all TCA cycle metabolites, which could indicate that unlabeled material present before the labeling period contained substantial succinate, or that the cells contain a stable intracellular succinate pool or generate succinate from other carbon sources.

### Glutamine increased the total abundance of all TCA metabolites except for succinate, which decreased

**Fig. 3:**
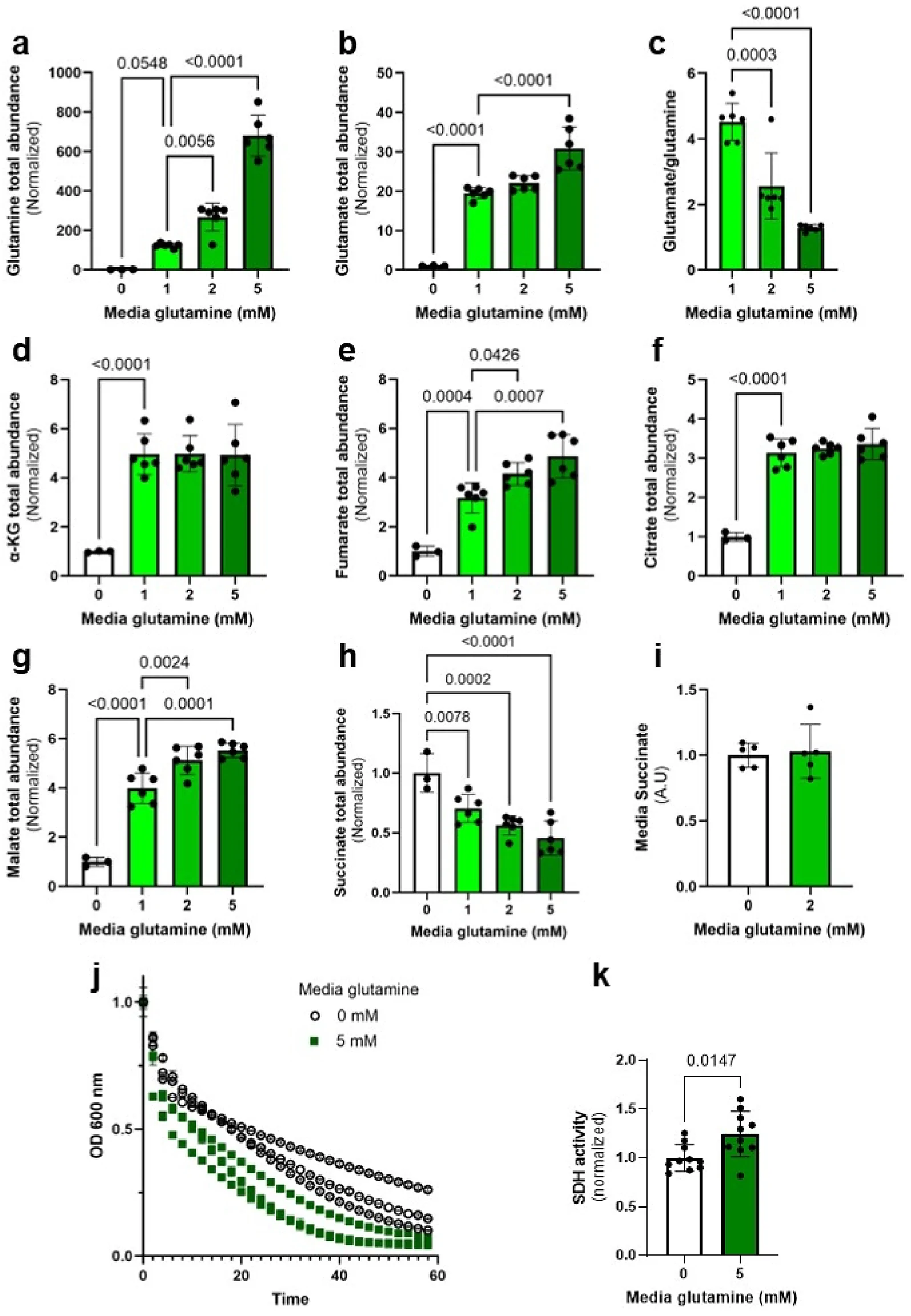
Increasing glutamine increased total abundance of all TCA metabolites except for succinate which decreased. HCAECs were treated with 0, 1, 2, or 5 mM of 1-^13^ C- or 5-^13^ C-glutamine for 24 h and then analyzed by LC-MS. **a**-**c** Total abundance of L-glutamine, L-glutamate and glutamate: glutamine ratio. Total abundance of TCA cycle metabolites (**d**) α-KG, **e** fumarate, **f** citrate, **g** malate, and **h** succinate. *n* = 6 samples for 1, 2, and 5 mM glutamine and *n* = 3 for 0 mM glutamine. All data points were normalized to the average value at 0 mM glutamine. Data were analyzed using ordinary one-way ANOVA with a Dunnett post-hoc multiple comparisons test. **i** Succinate was measured in media by LC-MS after 24 h HCAEC incubation with 0 or 2 mM glutamine. *n* = 5 biological replicates. **j** Representative curve and summation of (**k**) succinate dehydrogenase (SDH) activity for HCAEC cultured in 0 and 5 mM glutamine for 24 h. All data points were normalized to average value at 0 mM glutamine. *n* = 10 biological replicates. Data were analyzed using Mann Whitney test

Next, we analyzed the total abundance of glutamine, glutamate, and TCA metabolites by determining the integrated peak area, which is proportional to the concentration. For HCAEC in 2 or 5 mM glutamine, intracellular glutamine total abundance was 2 and 5 times higher than for HCAEC in 1 mM glutamine (*p* = 0.0056 and *p* < 0.0001, respectively; Fig. 3a). Intracellular glutamate total abundance was 20 times higher for HCAEC cultured in 1 mM glutamine as compared to 0 mM glutamine (*p* < 0.0001) and then an additional 50% higher for HCAEC in 5 mM glutamine as compared to 1 mM glutamine (*p* < 0.0001; Fig. 3b). The glutamate: glutamine total abundance ratio for HCAEC in 2 and 5 mM glutamine declined by 50% and 75%, respectively, as compared to the ratio for HCAEC in 1 mM glutamine (*p* = 0.0003, and < 0.0001, respectively; Fig. 3c). These total abundance data confirm that the endothelial cells continued to take up more glutamine as extracellular glutamine concentration increased but did not produce more glutamate, as previously shown (Fig. 1).

Similar to the labeled fraction data, TCA metabolite total abundance increased in the presence of extracellular glutamine, except for succinate, which decreased. α-KG, fumarate, citrate, and malate total abundance increased between 3 and 5 times for HCAEC cultured in 1 mM as compared to 0 mM glutamine (Fig. 3d-g). While α-KG and citrate total abundance did not change at higher glutamine concentrations, malate and fumarate demonstrated small increases with glutamine concentration. Malate total abundance was 35% higher at 2 mM glutamine (*p* = 0.0024) and 46% higher at 5 mM glutamine (*p* = 0.0001) as compared to 1 mM glutamine, and fumarate was 29% higher at 2 mM glutamine (*p* = 0.0426) and 51% higher at 5 mM glutamine (*p* = 0.0007) as compared to 1 mM glutamine. In contrast to the other TCA metabolites, succinate total abundance decreased by 34%, 44%, and 56% in HCAEC cultured in 1, 2, and 5 mM glutamine compared to 0 mM glutamine (*p* = 0.0078, 0.0002, and < 0.0001, respectively; Fig. 3h).

We then analyzed several mechanisms by which succinate total abundance could decrease with increasing glutamine. Cells can transport succinate out of the cell via MCT1 and OAT transporters (Bisbach et al., 2022; Huang et al., 2024). However, when we measured succinate in the media by LC-MS after 24 h of HCAEC incubation with 0 or 2 mM glutamine, we did not observe an increase in extracellular succinate (Fig. 3i). Glutamine deprivation has also been shown to cause deSUMOylation of succinate dehydrogenase (SDH), leading to reduced SDH activity and therefore reduced succinate oxidation to fumarate (Xia et al., 2021; Liu et al., 2023). We therefore measured how increasing extracellular glutamine affected SDH activity. SDH activity was 19% higher in HCAEC in 5 mM compared to 0 mM glutamine (*p* = 0.0117; representative experiment in Fig. 3j, three compiled experiments in Fig. 3k). Thus, increased SDH activity could be one means by which elevated extracellular glutamine reduced intracellular succinate.

###  Glutamine enriched amino acids and glutathione in addition to TCA metabolites

**Fig. 4:**
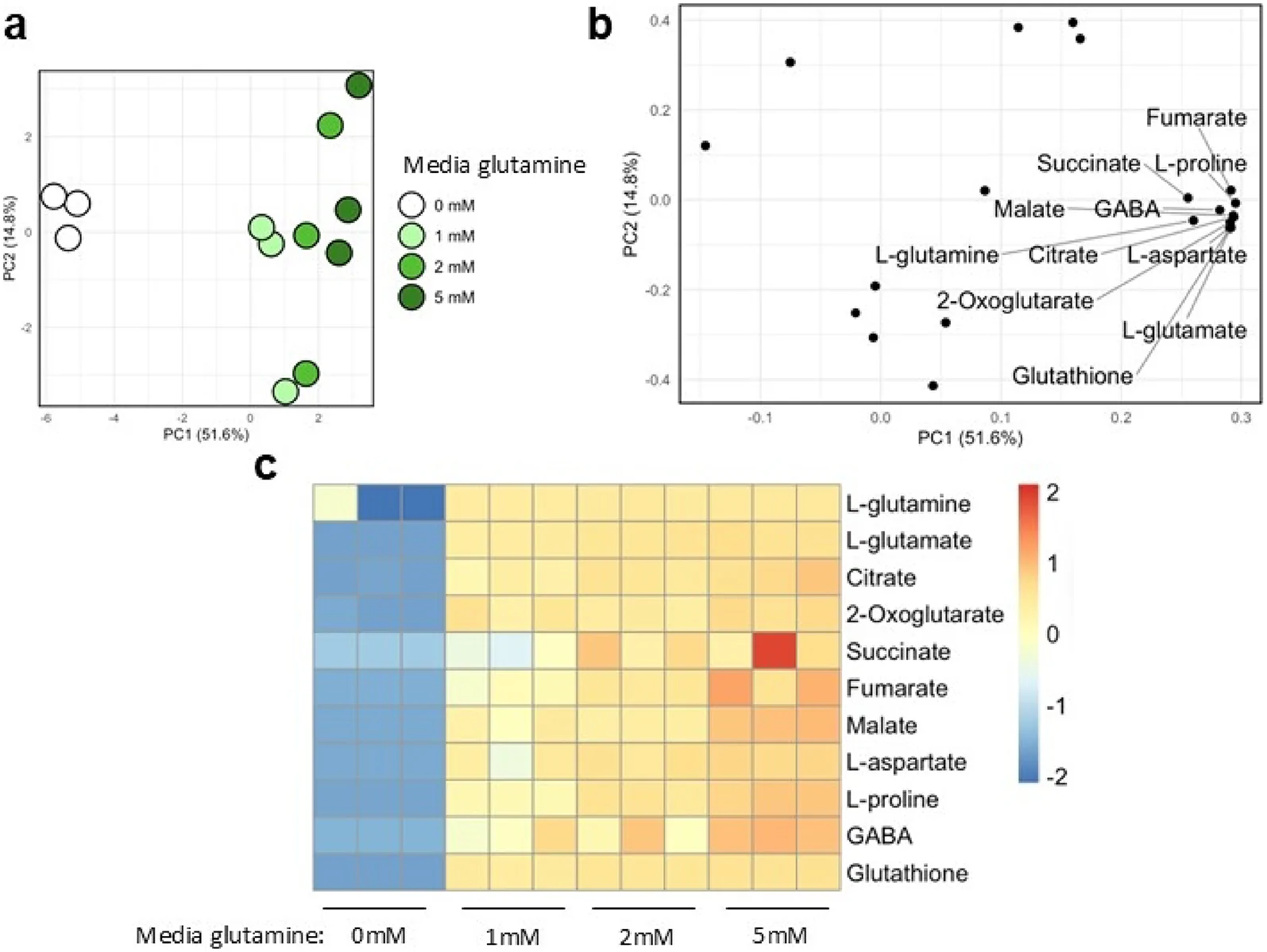
Glutamine carbons were incorporated into amino acids and glutathione in addition to TCA metabolites. HCAECs were cultured with 0, 1, 2, or 5 mM 5-^13^C-glutamine for 24 h, after which metabolites were extracted and profiled by LC–MS. **a** PCA score plot of the labeled fraction of each metabolite, showing a separation between cells cultured with versus without glutamine but minimal differences with increasing glutamine concentration. **b** PCA loading plot of the labeled fraction, with the top 11 loadings labeled. **c** Heatmap of metabolite labeled fractions

Since our data showed that ECs took up more glutamine as extracellular concentration increased but did not secrete more glutamate or proportionally increase TCA metabolite labeling, we investigated where the extra glutamine was metabolized using our LC-MS data. A principal component analysis (PCA) score plot of the 5-^13^ C-glutamine metabolite labeled fractions revealed clear separation of samples along component 1 (PC1 = 51.6%), with the 0 mM glutamine condition clustering distinctly from glutamine-treated groups (Fig. 4a). To identify the metabolites driving the separation observed in the PCA score plot, we next examined the PCA loading plot (Fig. 4b). In addition to glutamine, glutamate, and TCA cycle metabolites, the amino acids L-proline, L-aspartate, and GABA along with the tripeptide glutathione most contributed to the separation of glutamine-treated cells (Fig. 4b). A heatmap of the glutamine-labeled fractions of metabolites that most strongly contributed to the separation further indicated that HCAEC treated with 1, 2, or 5 mM glutamine are metabolically distinct from HCAEC treated with 0 mM glutamine but not highly different from each other (Fig. 4c). The PCA score plot of the 1-^13^ C-glutamine metabolite labeled fractions also showed that the 0 mM glutamine condition clustered distinctly from glutamine-treated groups (Online resource 5a), and the metabolites identified using the PCA loading plot were similar to those from the 5-^13^ C-glutamine metabolite (Online resource 5b, c).

While labeled fraction analysis provides information on isotopologue distribution, it does not capture changes in the overall metabolite pool size. To address this, we performed PCA on total metabolite abundance. The PCA score plot revealed separation along PC1 between the 0 mM glutamine condition and glutamine treated samples (Online resource 6). Notably, the 1 mM glutamine condition formed a distinct cluster from the 5 mM glutamine condition along PC1, indicating that although labeled fraction analysis did not show major differences among glutamine concentrations, total metabolite abundance was sensitive to increased glutamine. These results suggest that glutamine concentration influenced metabolic pool sizes even when isotopologue distributions appeared similar.

### Glutathione and proline were enriched with glutamine-derived carbons and increased in total abundance with glutamine; however, arginine and citrulline total abundance decreased with increasing glutamine

**Fig. 5:**
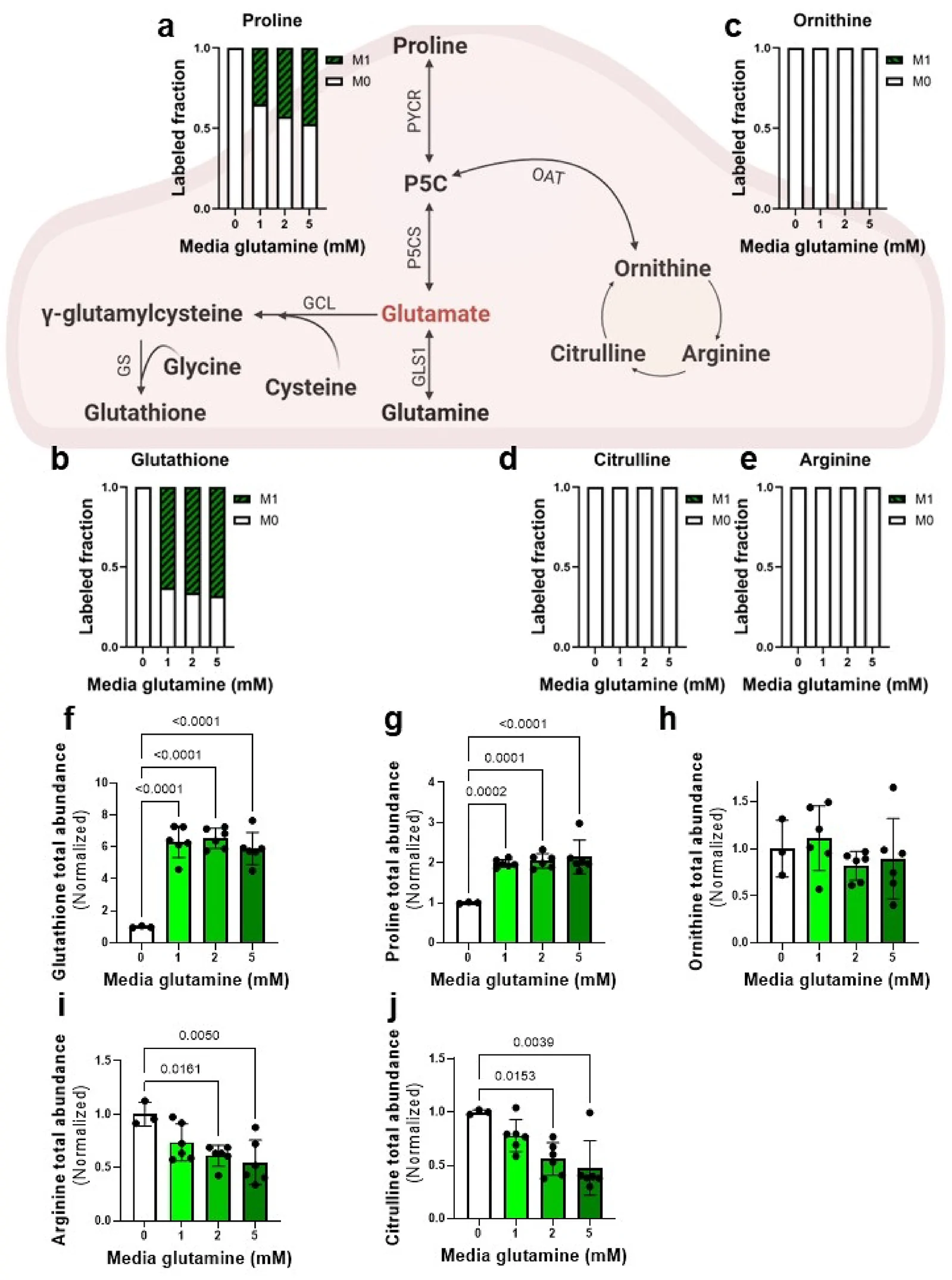
Glutathione and proline were enriched with glutamine-derived carbons. HCAECs were cultured with 0, 1, 2, or 5 mM 1-^13^C- or 5-^13^ C-glutamine for 24 h, after which cells were collected and the isotope distribution and total abundance of metabolites were determined using LC-MS. **a**-**e** Glutathione, proline, ornithine, citrulline, and arginine isotope labeled fractions. *n* = 6 biological replicates. Quantification of total abundance of **f**-**j** proline, glutathione, ornithine, citrulline, and arginine. *n* = 6 biologic replicates for 1,2,and 5 mM glutamine and *n* = 3 biologic replicates for 0 mM glutamine. Data were analyzed using ordinary one-way ANOVA with a Tukey post-hoc multiple comparisons test. Created in BioRender. Kheradmand-Hajibashi, M. (2026) https://BioRender.com/pb4r3y6

HCAEC cultured with glutamine separated from HCAEC cultured without glutamine due to glutamine-derived glutamate contributing carbon to both glutathione and proline. Glutathione isotopic enrichment from 1-^13^C-glutamine and 5-^13^C-glutamine increased from no detectable enrichment at 0 mM glutamine to 63% enrichment at 1 mM glutamine, respectively (Fig. 5a). Glutathione isotopic enrichment increased by an additional ~ 5% at 2 and 5 mM glutamine. Total glutathione abundance increased by more than 6 times when glutamine was increased from 0 mM to 1, 2, or 5 mM (*p* < 0.0001; Fig. 5f); however, no differences in total glutathione abundance were observed among HCAEC cultured in 1, 2, or 5 mM glutamine.

**Fig. 6:**
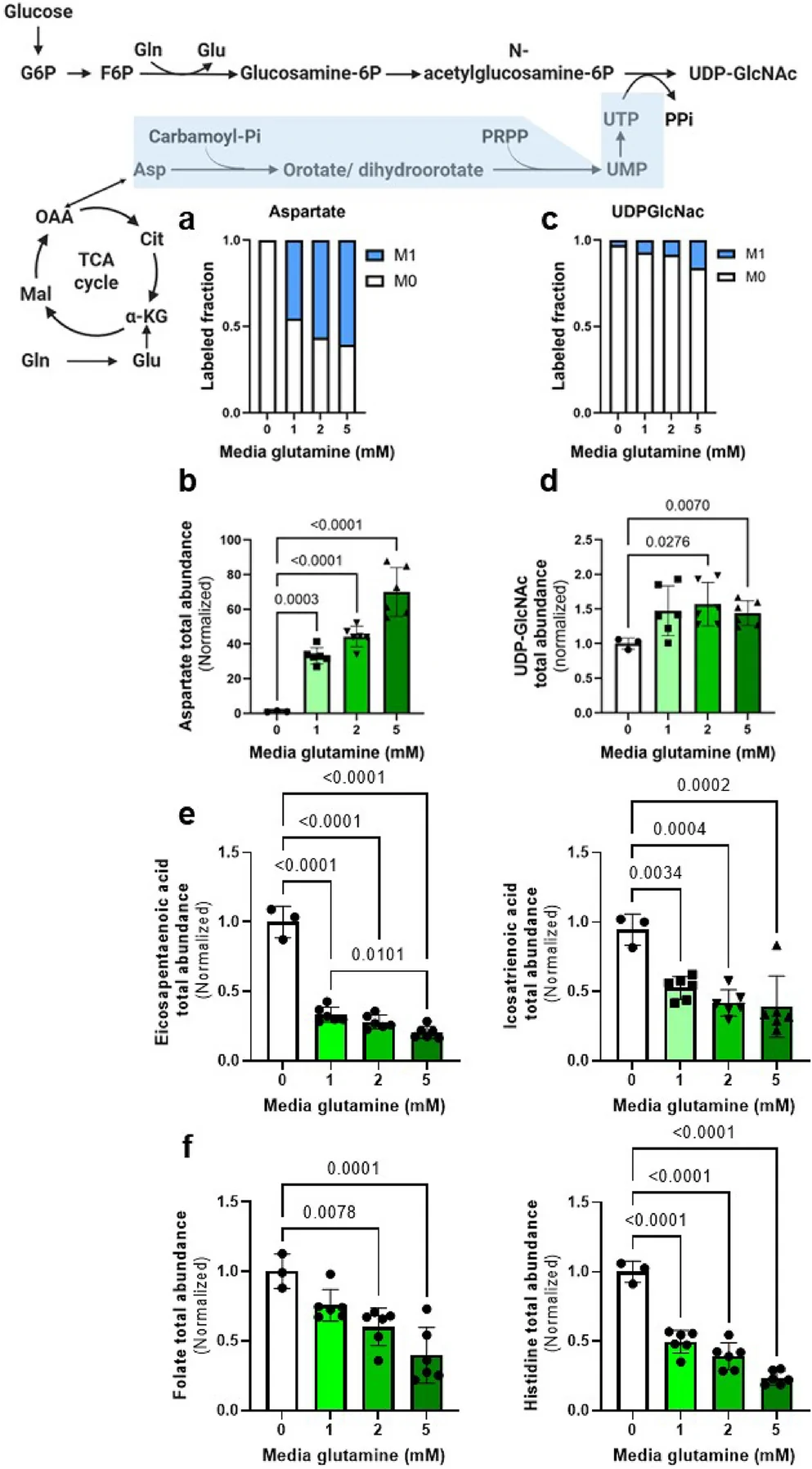
Glutamine-derived aspartate contributed carbons to UDP-GlcNAc, and glutamine altered fatty acids and one-carbon metabolism. HCAECs were treated with 0, 1, 2, and 5 mM 1-^13^C- or 5-^13^ C-glutamine for 24 h, after which cells were collected, and the isotope enrichment and total metabolite abundance was determined using LC-MS. Schematic of glutamine-derived aspartate incorporation into UDP-GlcNAc. Isotope enrichment from 5-^13^ C-glutamine into (**a**) aspartate, and (**c**) UDP-GlcNac. *n* = 3 biological replicates. Quantification of total abundance of (**b**) aspartate, and (**d**) UDP-GlcNAc, (**e**) eicosapentaenoic acid (EPA) and icosatrienoic acid and (**f**) one carbon metabolites folate and histidine. *n* = 6 biologic replicates for 1, 2, and 5 mM glutamine and *n* = 3 biologic replicates for 0 mM glutamine. Data were analyzed using ordinary one-way ANOVA with a Tukey post-hoc multiple comparisons test. Created in BioRender. Kheradmand-Hajibashi, M. (2026) https://BioRender.com/xjjzmwd

Glutamate can also be metabolized to pyrroline-5-carboxylate (P5C), which can then be directed toward either proline synthesis or the ornithine cycle. Proline isotopic enrichment increased by 36%, 43%, and 48% at 1, 2, and 5 mM 1-^13^C-glutamine and 5-^13^C-glutamine, respectively, compared to 0 mM glutamine (Fig. 5b). Proline total abundance doubled in HCAEC cultured in 1 mM glutamine as compared to 0 mM glutamine (Fig. 6g; *p* = 0.0002). Similar to glutathione, no difference in proline total abundance was observed in HCAEC cultured in 1, 2, and 5 mM glutamine. In contrast, there was no detectable carbon enrichment in ornithine cycle metabolites (Fig. 5c-e). However, total metabolite abundance in this pathway was altered by glutamine (Fig. 5h-j). Increasing extracellular glutamine from 0 to 2 or 5 mM decreased citrulline total abundance by 47% (*p* = 0.0153) and 53% (*p* = 0.0039), respectively. Similarly, increasing glutamine decreased arginine total abundance by 39% at 2 mM glutamine (*p* = 0.0161) and 46% at 5 mM glutamine (*p* = 0.0050) compared to 0 mM glutamine. Ornithine total abundance remained unchanged across glutamine concentrations. Together, the isotopic enrichment data suggest that glutamine contributes carbon to glutathione and proline but not to ornithine cycle metabolites. Reduced arginine and citrulline abundance suggest that glutamine altered their utilization.

### Glutamine contributed carbons to UDP-GlcNAc via aspartate and altered fatty acids and one-carbon metabolism

Since we also observed aspartate isotope enrichment, we next examined how increasing extracellular glutamine concentration impacted pathways downstream of aspartate such as UDP-GlcNAc synthesis. Glutamine-derived α-ketoglutarate becomes oxaloacetate, which is then transaminated to form aspartate. Aspartate is incorporated into orotate, which becomes uridine triphosphate (UTP) and is then incorporated into UDP-GlcNAc (Fig. 6). LC-MS analysis showed increased isotopic enrichment of aspartate from 5-^13^C-glutamine, with enrichment increasing by 45%, 56%, and 60% at 1, 2, and 5 mM glutamine, respectively, compared to no glutamine (Fig. 6a). Aspartate total abundance increased by 33-, 44-, and 70-fold as glutamine increased from 0 mM to 1, 2, and 5 mM, respectively (*p* = 0.0003, < 0.0001; Fig. 6b). Isotopic distribution analysis from 5-^13^C-glutamine revealed a progressive increase in labeled carbon incorporation into UDP-GlcNAc, with enrichment increasing by 7%, 9%, and 16% at 1, 2, and 5 mM glutamine, respectively (Fig. 6c). In parallel, total UDP-GlcNAc abundance increased by around 50% as glutamine concentration increased from 0 to 2 or 5 mM glutamine (*p* = 0.0276, and 0.0070, respectively; Fig. 6d), with no differences observed between 2 and 5 mM glutamine. Collectively, the isotope enrichment data suggests that increasing glutamine increases its incorporation into UDP-GlcNAc through increased aspartate availability.

We observed that the total abundance of unsaturated fatty acids eicosapentaenoic acid (EPA) and icosatrienoic acid decreased with increasing glutamine concentration. EPA abundance decreased by 70% for HCAEC in 1 mM glutamine as compared to 0 mM glutamine (*p* < 0.0001, Fig. 6e) and decreased an additional 12% for HCAEC in 5 mM glutamine (*p* = 0.0101). Similarly, icosatrienoic acid abundance decreased by 48% for HCAEC in 1 mM glutamine as compared to 0 mM glutamine (*p* = 0.0034, Fig. 6e), with no additional decrease at higher glutamine concentrations.

We finally observed significant alterations in the total abundance of metabolites associated with 1 C metabolism, a process that supports purine biosynthesis, DNA and protein methylation, and redox homeostasis among others (Ducker & Rabinowitz, 2017; Hwang et al., 2023). In the folate cycle, which supports 1 C metabolism, the biologically active form of folate (tetrahydrofolate, THF) and its reduced form (5-methyl-THF) serve as carriers for 1 C units. New 1 C units enter the folate cycle from the amino acids serine, glycine, and histidine among others. In our LC-MS data, increasing glutamine concentration from 0 to 2 mM decreased total folate abundance by 25% (*p* = 0.0078), while increasing glutamine concentration from 0 to 5 mM decreased total folate abundance by 60% (*p* = 0.0001, Fig. 6f). Similarly, increasing glutamine concentration from 0 to 1, 2, or 5 mM decreased total histidine abundance by 51%, 61%, or 77% (*p* < 0.0001 for all). Both glycine and serine total abundance showed smaller changes with increasing glutamine, with glycine 38% lower at 5 mM glutamine compared to 1 mM glutamine (*p* = 0.01870, online resource. 7). These total abundance data suggest that glutamine altered 1 C metabolism.

## Discussion

Glutamine supplementation shows potential both *in vitro* and *in vivo* to preserve endothelial function under diverse stress conditions. However, how extracellular glutamine reprograms the endothelial metabolic network remains poorly defined. In this study, we systematically examined the metabolic fate of glutamine in endothelial cells over increasing glutamine concentrations (0 to 10 mM). We showed that endothelial cells increase glutamine uptake as extracellular glutamine concentration increases, but glutamate secretion and oxidative respiration saturate above 2 mM glutamine, suggesting a limited capacity for glutaminolysis. Glutamine’s carbons primarily enter the forward TCA cycle, with a small contribution to reverse carboxylation. Glutamine carbons then support glutathione and non-essential amino acid synthesis. Although most TCA metabolite abundance increased with extracellular glutamine, succinate, unsaturated fatty acid, and 1 C metabolite total abundance declined. Together, these findings indicate that while endothelial cells take up more glutamine when it is available, excess glutamine is stored rather than metabolized. The presence of glutamine, but not its excess, sustains the TCA cycle, amino acid synthesis, and redox balance.

In both normal and high glucose, glutamine uptake and intracellular glutamine increased with extracellular glutamine concentration. While others have shown that increasing extracellular glutamine from 0 to 2 mM increased intracellular glutamine, for example in bovine venular endothelial cells (Meininger & Guoyao, 1997) and in human diploid fibroblasts (Bannai & Ishii, 1988), our study is the first to our knowledge to examine the extremely high glutamine concentrations often used in *in vitro* cell culture. Excess glutamine uptake would come at a cost to the endothelial cell, given that the primary glutamine importer ASCT2 (SLC1A5) imports sodium and exports a neutral amino acid (e.g., serine, threonine). Glutamine uptake would increase energy use, since the cell must use the Na⁺/K⁺ ATPase to maintain the sodium gradient. This excess energy likely comes from oxidative respiration, since we did not observe an increase in glycolysis. The net export of amino acids by ASCT2 would also need to be countered by amino acid import from system A and N transporters (SNAT1-5).

In contrast, intracellular glutamate and glutamate secretion plateaued above 2 mM glutamine, suggesting that excess glutamine is stored rather than metabolized. Intracellular glutamate saturation may occur due to limitations in its production, while extracellular glutamate saturation may occur due to limitations in its export. Glutamine is metabolized to glutamate by GLS-1. We measured an increase in GLS-1 as glutamine increased from 0 to 0.5 mM but no further increase up to 2 mM glutamine. Furthermore, intracellular glutamate binds to and reduces GLS-1 activity (Shapiro et al., 1982; Cyriac & Lee, 2024). Thus, GLS-1 availability and activity may limit glutamine conversion to glutamate despite continued glutamine uptake. The observed extracellular glutamate plateau may reflect limits in amino acid exchange. Glutamate export is functionally coupled to cystine uptake through the cystine–glutamate antiporter xCT (Jyotsana et al., 2022). In human diploid fibroblasts, extracellular glutamate increased with extracellular cystine, indicating that extracellular cystine is essential for glutamate efflux (Bannai & Ishii, 1988). Therefore, glutamine-derived intracellular glutamate could be exported in exchange for extracellular cystine up to the point at which cystine availability becomes limiting. These data suggest that glutamine supplementation alone may not be sufficient to fully exploit the protective effects of glutamine, including enhanced antioxidant capacity and inflammation reduction (Jiang et al., 2017; Peyton et al., 2018; Kheradmand et al., 2026). Increasing GLS-1 expression or activity or promoting glutamate efflux through increased extracellular cystine may be required.

Glutamine availability may support elevated endothelial metabolic activity, since we observed increased oxidative respiration without a decrease in glycolysis. Indeed, glutamine supplementation actually increased lactate secretion. This may occur because glutamine provides carbons to the TCA cycle to support oxidative respiration. Glucose-derived pyruvate can then be reduced to lactate and secreted, rather than oxidized in the TCA cycle. Alternatively, glutamine could act as a signaling metabolite that activates mechanistic target of rapamycin (mTOR) and thereby promotes glycolytic metabolism (Szwed et al., 2021). Further experiments are needed to confirm this observation and determine the impact of glutamine-induced elevated metabolism on endothelial function.

Our isotope enrichment data confirmed work by others showing that glutamine is a major mitochondrial substrate for endothelial cells (Huang et al., 2017; Kim et al., 2017; Kaczara et al., 2024). Parallel labeling with 1-^13^C- and 5-^13^C-glutamine showed that endothelial cells metabolize glutamine primarily in the forward oxidative TCA cycle but also in reductive carboxylation. Oxidative glutamine metabolism supports the production of ATP and TCA intermediates for energy production, while reductive glutamine metabolism supports the production of citrate and acetyl-CoA for lipid synthesis (Metallo et al., 2012). Interestingly, excess glutamine did not change the percentage of glutamine that underwent reductive carboxylation, suggesting that glutamine does not shift endothelial cell energy vs. biomass requirements or affect mitochondrial activity. Instead, reductive carboxylation seems to be driven by hypoxia. In the A549 cell line, reductive carboxylation increased from ~ 10% to 80% in hypoxia (Metallo et al., 2012). We measured lower glutamine reductive carboxylation than in other studies (5% vs. 13%) (Kim et al., 2017), which could relate to variations in cell type (HUVEC vs. HCAEC) or nutrient and oxygen availability.

In contrast to other TCA metabolites, which showed increased total abundance as extracellular glutamine increased, succinate total abundance decreased as glutamine concentration rose. We measured a large unlabeled intracellular succinate pool in our HCAEC, which is consistent with studies in HUVEC and iPSC-derived brain microvascular endothelial cells (Moiz et al., 2021; Weber et al., 2025). In HepG2 cells, glutamine deprivation promoted deSUMOylation of succinate dehydrogenase substrate A (SDHA), impairing SDH assembly and activity (Liu et al., 2023). Consistent with this, our data showed that glutamine enhanced SDH activity, which likely increased succinate consumption and contributed to the reduced intracellular succinate pool. However, the change in succinate could also be caused by reduced succinate production, altered compartmentalization, changes in succinate turnover, or dilution (Abdullah et al., 2023). These alternative mechanisms would also need to be tested via targeted or dynamic isotope tracing.

We and others demonstrated that glutamine reduces intracellular oxidative stress through GSH synthesis (Peyton et al., 2018; Kheradmand et al., 2026). However, increasing glutamine above 1 mM did not further increase GSH total abundance. The first step of GSH synthesis, in which glutamate and cysteine are combined in a reaction catalyzed by glutamate cysteine ligase (GCL), is rate-limiting and may explain the plateau in GSH production (Zhang & Forman, 2012; Li et al., 2022). The glutamate concentration at which this reaction rate is half of its maximum velocity (K_m_) is 1.8 mM, which is much lower than the estimated intracellular glutamate concentration (~ 21 mM in fibroblasts). Thus, GCL is likely already saturated with glutamate, so additional glutamine-derived glutamate would not increase GSH production (Lu, 2013). Furthermore, GSH itself binds to the glutamate site on GCL to inhibit its activity (K_i_=2.3 mM), meaning that high GSH can also reduce GSH production (Richman & Meister, 1975; Franklin et al., 2009). Thus, high extracellular glutamine alone is not sufficient to increase endothelial GSH.

Ornithine cycle metabolites were not labeled with glutamine-derived carbons, but both citrulline and arginine decreased in abundance with extracellular glutamine. Ornithine aminotransferase (OAT) catalyzes the reversible reaction between P5C and ornithine. Given the lack of ^13^C labelled ornithine, this reaction appears to be primarily in the direction of P5C production from ornithine in our endothelial cells. Reduction of citrulline and arginine abundance may be explained by inhibited citrulline transport and reduced citrulline to arginine conversion. In bovine endothelial cells, extracellular glutamine reduced intracellular citrulline by inhibiting citrulline transport, likely because they share some of the same neutral amino acid transporters (Wu & Meininger, 1993). Furthermore, 2 mM glutamine inhibited arginine production from citrulline via argininosuccinate in bovine aortic endothelial cells (Sessa et al., 1990). Some studies also suggest that cultured endothelial cells do not have detectable carbamoyl-phosphate synthase I (CPS-I) activity, which is essential to transform ornithine into arginine (Wu et al., 2000).

The observed increase in UDP-GlcNAc abundance and isotopic enrichment from 5-^13^C-glutamine shows that glutamine and its carbons contribute to the endothelial cell HBP. Glutamine provides a nitrogen to convert fructose-6-phosphate into glucosamine-6-phosphate early in the HBP (Kornfeld, 1967; Wellen et al., 2010; Yang et al., 2026), and glutamine abundance correlates with UDP-GlcNAc synthesis (Paneque et al., 2023). However, UDP-GlcNAc synthesis integrates many metabolic inputs, one of which is uridine supplied by pyrimidine nucleotide metabolism (Bond and Hanover 2015; Paneque et al. 2023; Yang et al. 2024b). We observed increased aspartate total abundance and isotopic enrichment, suggesting that glutamine-derived aspartate contributes to UDP-GlcNAc formation (Oberkersch et al., 2022).

Finally, increasing glutamine decreased folate and histidine abundance, suggesting altered 1 C metabolism flux. 1 C metabolism is essential for nucleotide and amino acid biosynthesis, methylation reactions, and redox balance (Ducker & Rabinowitz, 2017; Petrova et al., 2023). In the 1 C pathway, folate serves as the central carrier by accepting and transferring single-carbon units. Histidine and serine contribute 1 C units to folate in the cytosol, while serine and glycine contribute 1 C units to folate in mitochondria (Ducker & Rabinowitz, 2017; Lin et al., 2022). The altered 1 C metabolism flux could relate to glutamine-induced enhanced cell proliferation via nucleotide synthesis (Peyton et al., 2018) or to enhanced antioxidants (Kheradmand et al., 2026). Additional experiments would be required to investigate this mechanism.

While our study shows the impact of glutamine on endothelial cells in culture, it is not without limitations. We treated endothelial cells at confluence, when endothelial cells are predominantly quiescent; however, we did not directly control and assess cell cycle phase. Because metabolic fluxes and isotopic labeling patterns differ between proliferating and quiescent endothelial cells (Kalucka et al., 2018), it would be interesting to compare glutamine metabolism across defined cell cycle states. In addition, while stable isotope tracing provided insight into glutamine carbon use in the forward and reductive carboxylation, the use of single-position ¹³C tracers limited our ability to resolve all possible carbon and nitrogen entry routes into downstream pathways. Finally, our analyses were performed at isotopic steady state following 24 h incubations and therefore reflect net metabolic outcomes rather than dynamic flux changes. Dynamic tracing experiments would be useful to capture transient metabolic reprogramming with glutamine availability.

## Conclusions

In summary, our work expands upon prior studies to define how increasing extracellular glutamine reprograms endothelial metabolism across a higher than physiological range, including concentrations commonly used in cell culture media. Using isotope assisted metabolomics, we show that endothelial cells increase glutamine uptake with increased availability, while glutamate secretion and glutaminolysis saturated above 2 mM glutamine. Glutamine increased isotopic enrichment and total abundance of most TCA cycle metabolites, glutathione, proline, aspartate, and UDP-GlcNAc, while reducing total abundance of succinate, citrulline, arginine, PUFAs, folate, and histidine. Together, these findings highlight glutamine as a metabolic regulator that maintains the endothelial TCA cycle and endothelial redox homeostasis.

## Supplementary Information

Below is the link to the electronic supplementary material.

Supplementary Material 1

Supplementary Material 2

## Data Availability

All data supporting the findings of this study are available within the article and its Supplementary Information files. Raw isotopomer and metabolomics datasets are provided in the Supplementary Data.
