# Supplementary material for: Excess glutamine rewires endothelial cell metabolism
Source: Metabolomics. 2026 Aug 22;22(5):143. doi: 10.1007/s11306-026-02515-4 (PMC13499863; doi:10.1007/s11306-026-02515-4)
Supplement: Supplementary file 1 [file 11306_2026_2515_MOESM2_ESM.pdf]

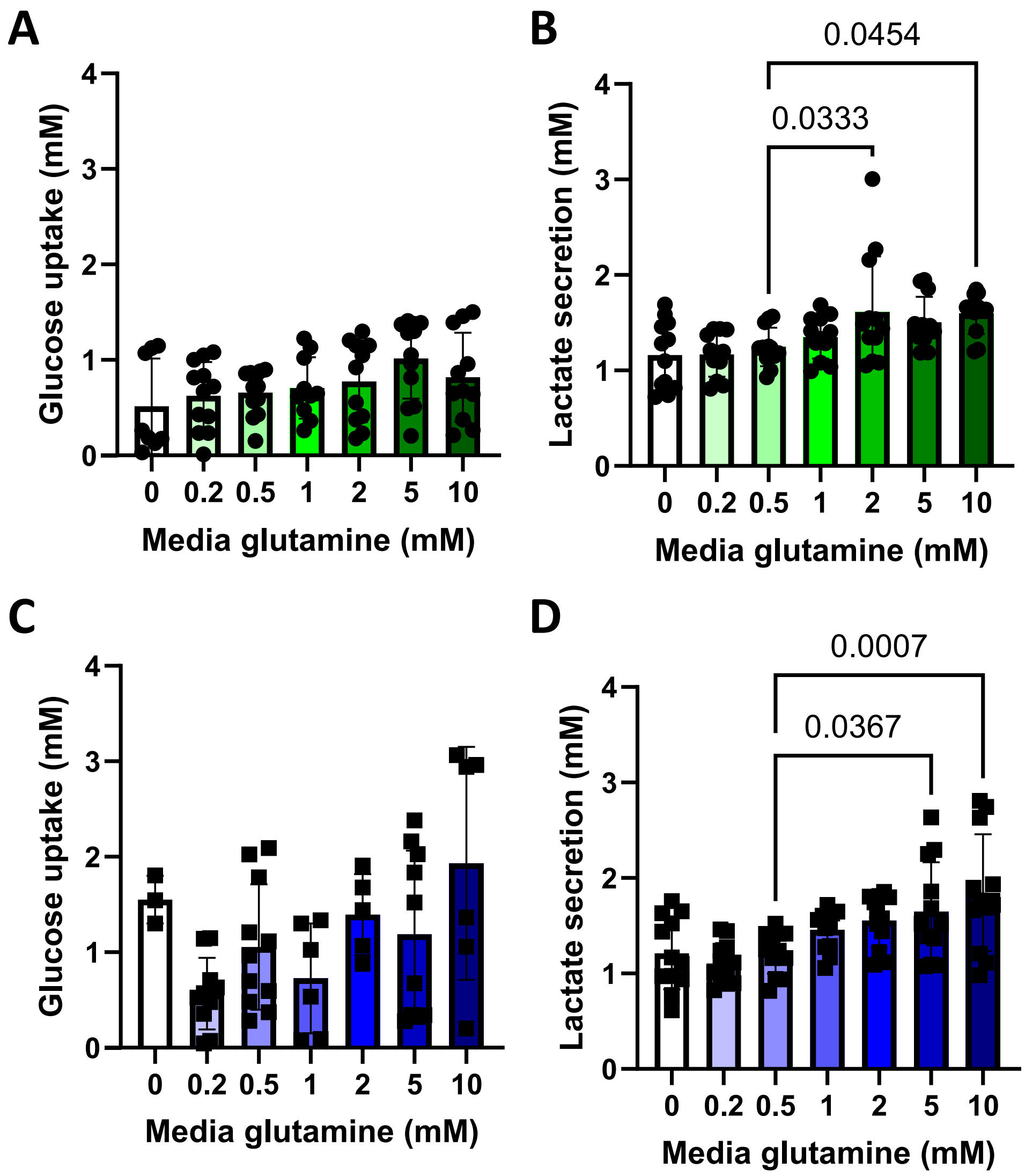

Supplementary Figure 1. Extracellular glutamine increased lactate secretion without affecting glucose uptake. HCAEC were incubated with 0-10 mM glutamine in normal (5.5) and high glucose (15 mM) for 24 hours. Glucose uptake and lactate secretion were measured in normal (a, b) and high (d, e) glucose using a YSI bioanalyzer. n=3-12 biological replicates. Data were analyzed using an ordinary one-way ANOVA with a Dunnett post-hoc multiple comparisons test, following an ROT outlier test.

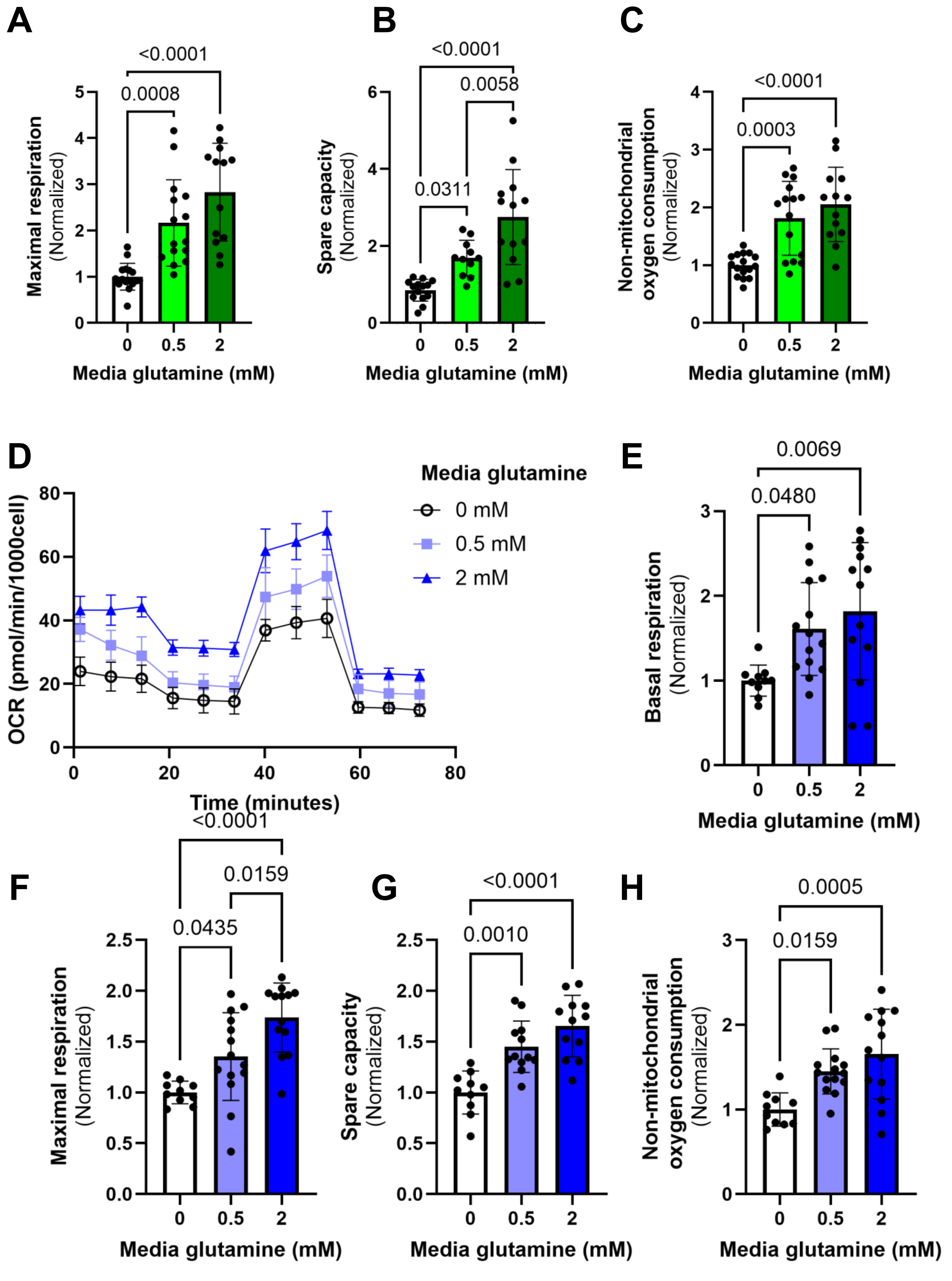

Supplementary Figure 2. Glutamine increased endothelial oxidative respiration in high glucose. HCAECs were incubated for 24 hours with 0, 0.5, or 2 mM glutamine in normal (5.5 mM) or high (15 mM) glucose culture. (A) maximal respiration, (B) spare capacity, and (C) non-mitochondrial oxygen consumption for normal glucose from three experiments, normalized to the 0 mM glutamine condition. (D) Representative oxygen consumption rate (OCR) as measured by Seahorse Mito Stress test for high glucose. (E) Basal respiration, (F) maximal respiration, (G) spare capacity, and (H) non-mitochondrial oxygen consumption from three experiments, normalized to the 0 mM glutamine. n=10-16 samples. Data were analyzed using an ordinary one-way ANOVA with a Tukey post-hoc multiple comparisons test.

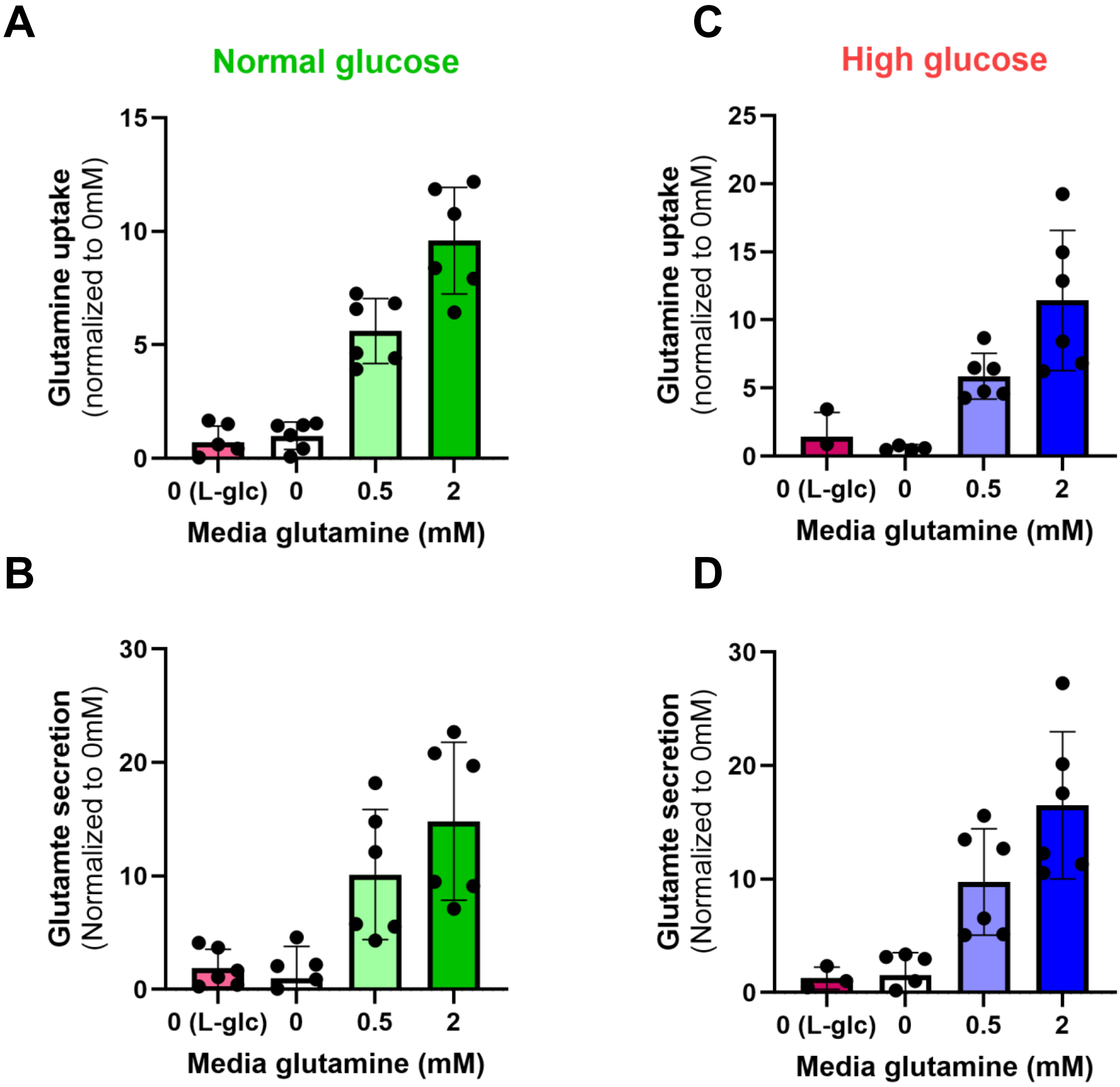

Supplementary Figure 3. Osmolarity did not impact EC glutamine uptake in normal and high glucose. L-glucose was added to the cell culture media as osmotic control for 2 mM glutamine in normal and high glucose. (A) Glutamine uptake and (B) glutamate secretion in normal glucose, (C) glutamine uptake and (D) glutamate secretion in high glucose, as measured via YSI. Data were normalized to the average glutamine uptake or glutamate secretion at 0 mM glutamine in each condition. n=6 samples.

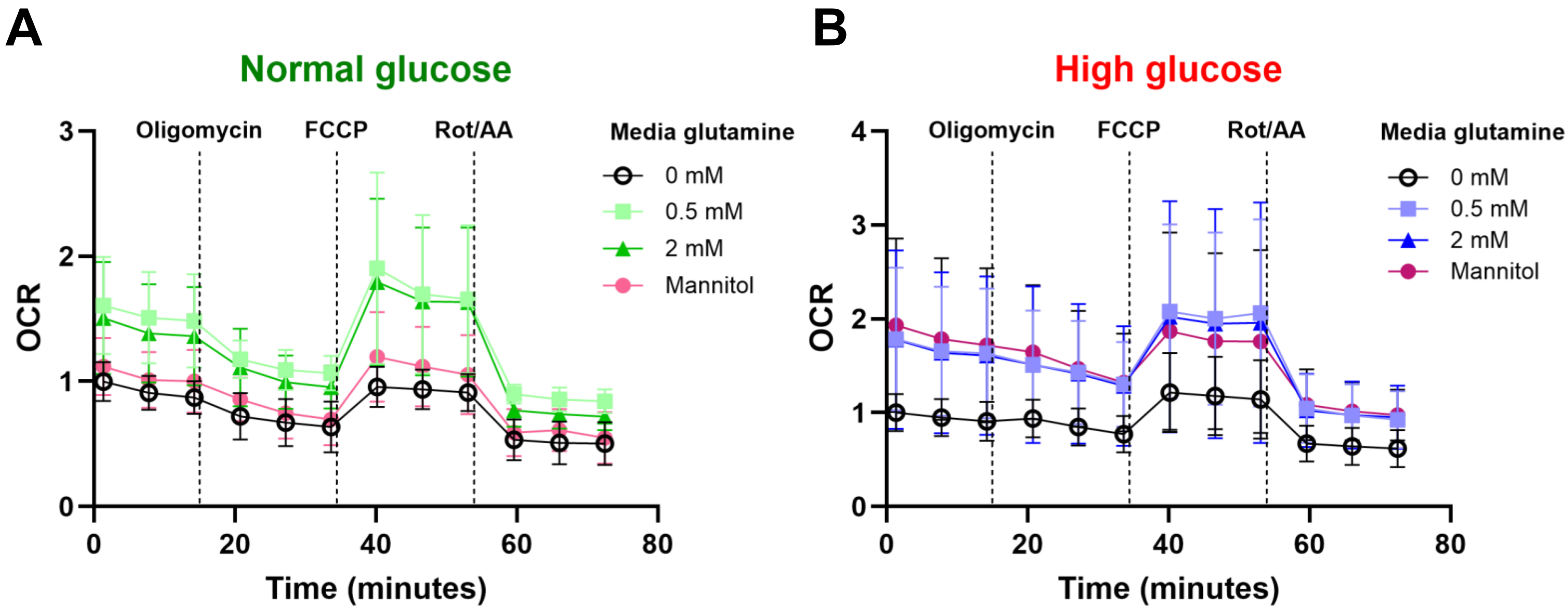

Supplementary Figure 4. Osmolarity increased OCR for HCAEC in high glucose. Cells were incubated in 0-2 mM glutamine in (A) normal (5.5 mM) and (B) high glucose (15 mM), including L-glucose as osmotic control (OC), for 24 hours before measuring OCR with a Seahorse Mito Stress Test. Data were normalized to the average OCR for cells in normal or high glucose with no glutamine. n=20 samples.

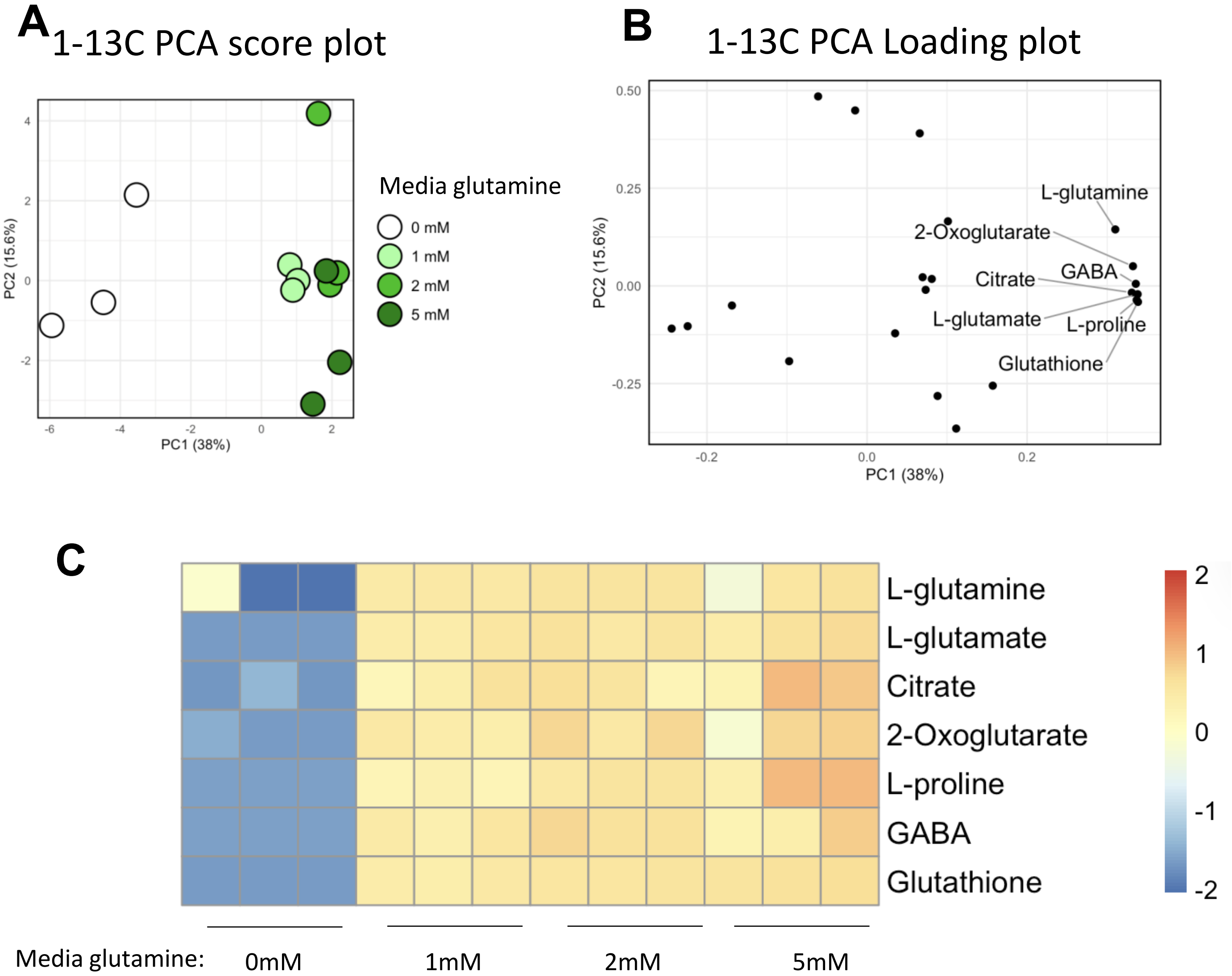

Supplementary Figure 5. 1-^{13}C-glutamine carbons were incorporated into citrate through the reverse TCA cycle, as well as glutathione and L-proline. HCAECs were cultured with 0, 1, 2, or 5 mM 1-^{13}C-glutamine for 24 hours, after which metabolites were extracted and profiled by LC–MS. (A) PCA score plot of the labeled fraction of each metabolite, with the top 7 loadings labeled. (B) PCA loading plot of the labeled fraction. (C) Heatmap of metabolite labeled fractions. n=3 samples

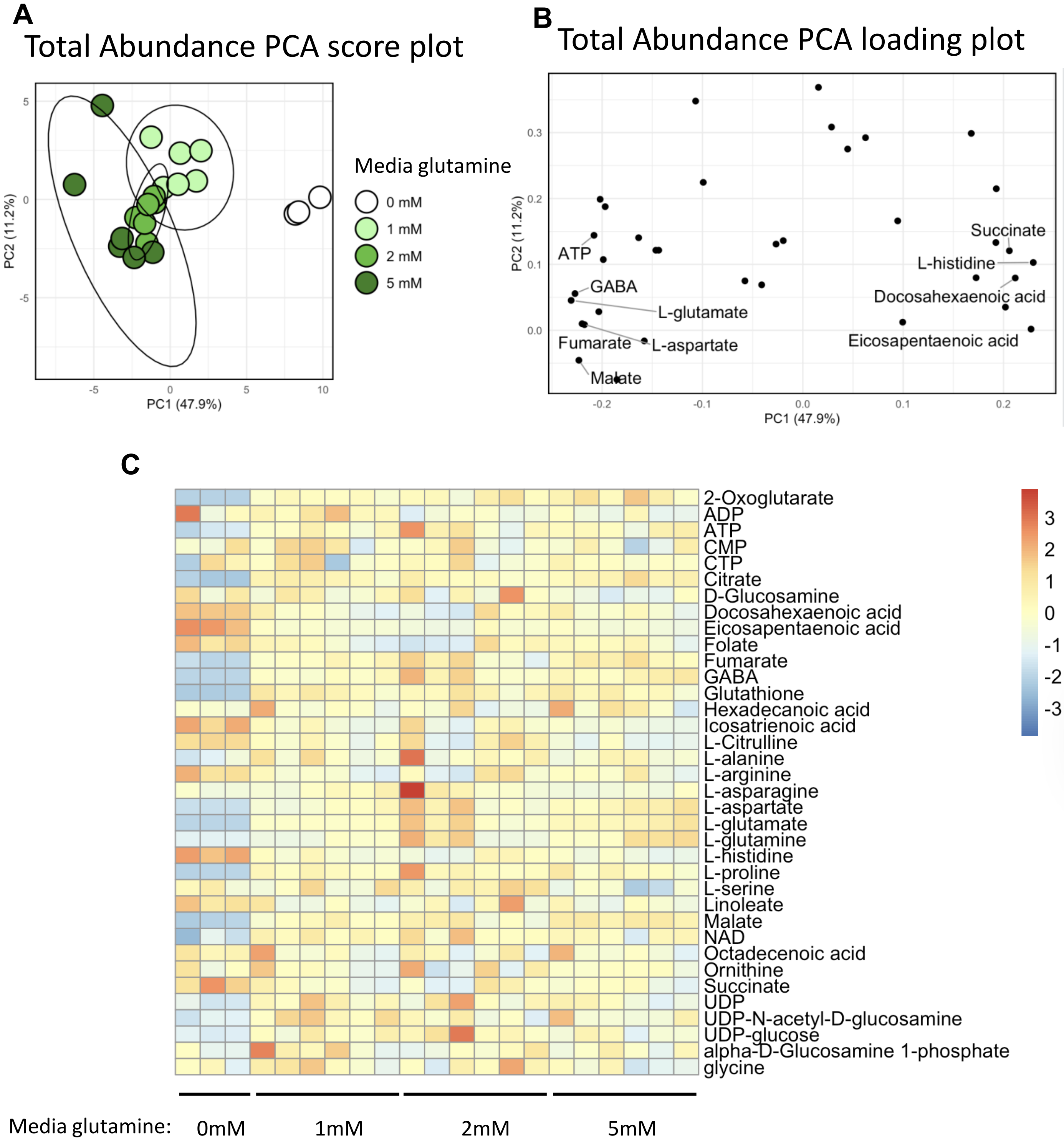

Supplementary Figure 6. Extracellular glutamine increased the intracellular pool of many TCA cycle metabolites but decreased the intracellular pool of succinate, histidine, and several fatty acids. HCAECs were cultured with 0, 1, 2, or 5 mM 1-^{13}C-glutamine for 24 hours, after which metabolites were extracted and profiled by LC–MS. (A) PCA score plot of the total abundance of each metabolite. (B) PCA loading plot of the total abundance of each metabolite, with the top10 loadings labeled. (C) Heatmap of metabolite labeled fractions.. n=3 samples for 0 mM glutamine; n=6 samples for 1 mM, 2 mM, and 5 mM glutamine.

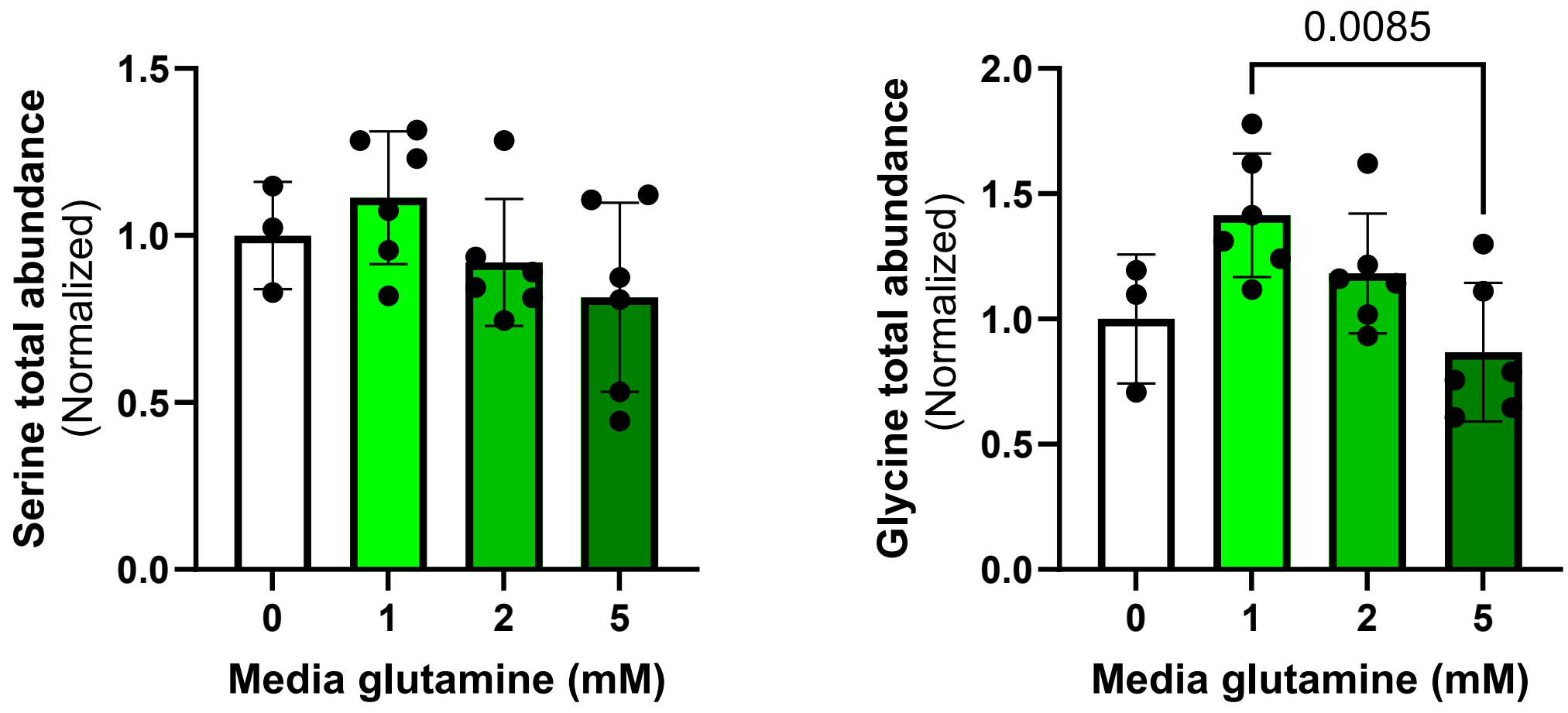

Supplementary Figure 7. Glutamine altered one-carbon metabolism. HCAECs were treated with 0, 1, 2, and 5 mM 1-^{13}C- or 5-^{13}C-glutamine for 24 hours, after which cells were collected, and the total metabolite abundance was determined using LC-MS. One carbon metabolites serine and glycin. n=6 samples for 1, 2, and 5 mM glutamine and n=3 samples for 0 mM glutamine. Data were analyzed using ordinary one-way ANOVA with a Tukey post-hoc multiple comparisons test.

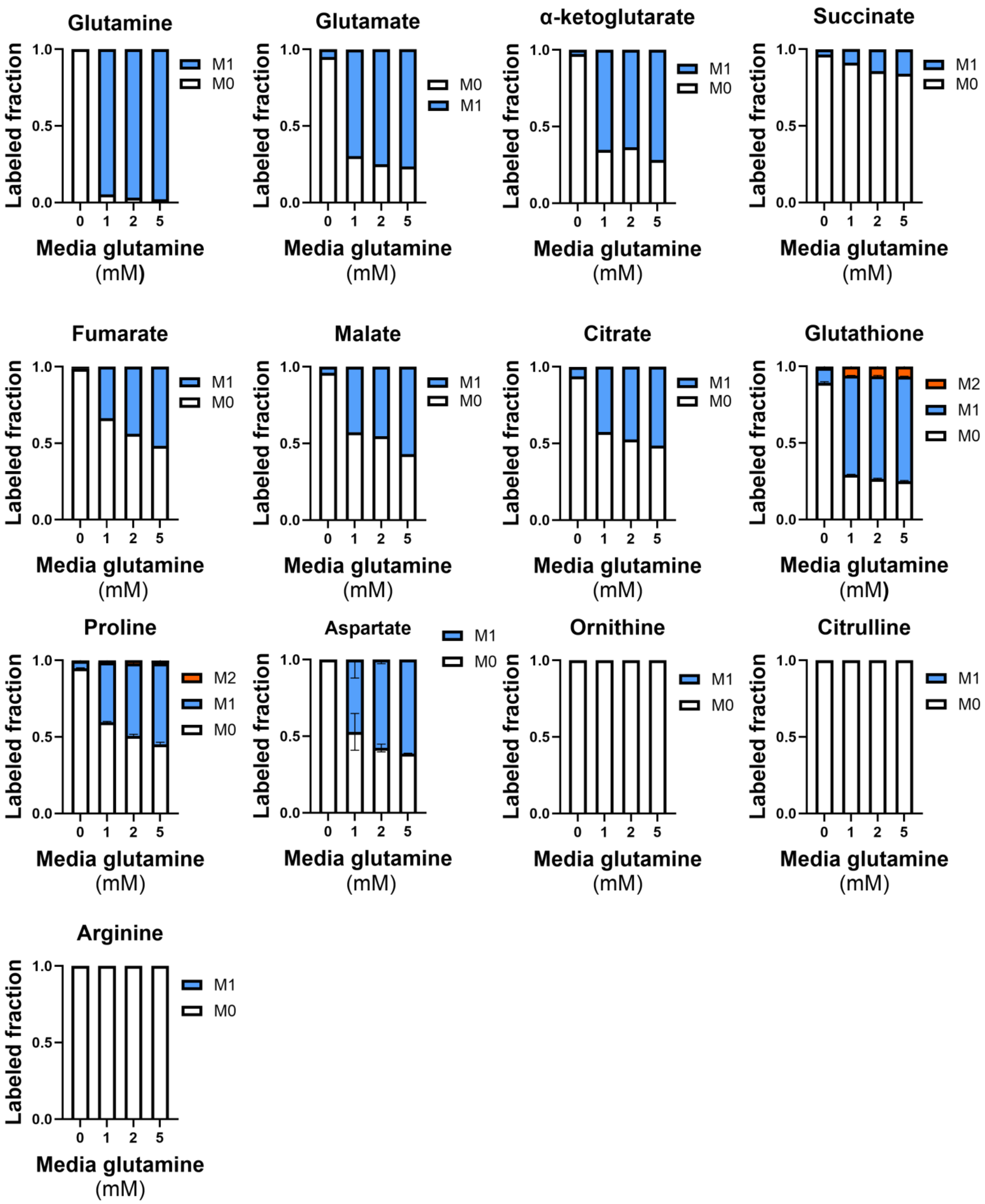

Supplementary Figure 8. Uncorrected mass distribution data from 5-13C-glutamine. HCAECs were cultured with 0, 1, 2, or 5 mM of 5-^{13}C-glutamine for 24 hours, after which cells were collected, and the isotope distribution was determined using LC-MS.n=3 biological replicates.

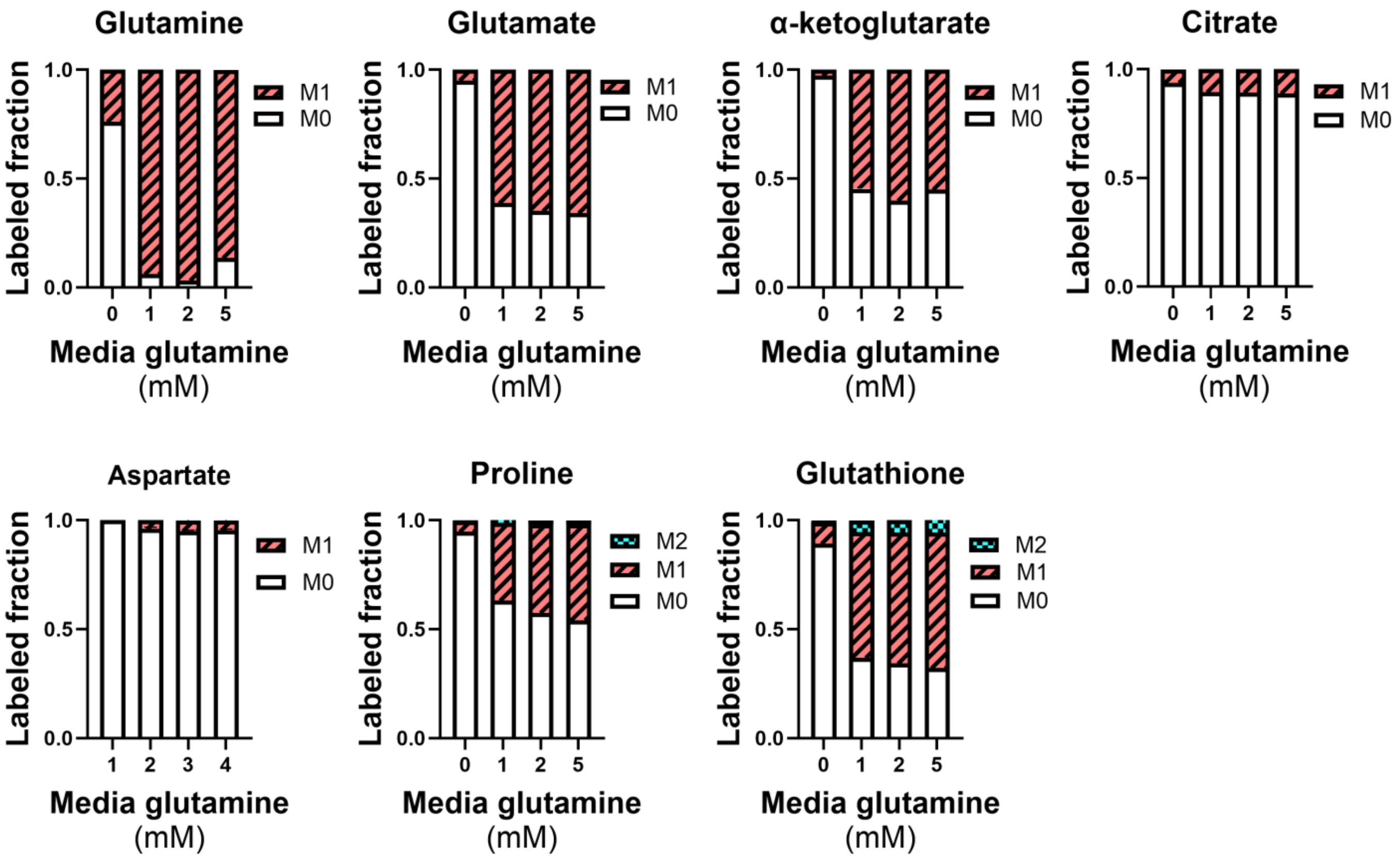

Supplementary Figure 9. Uncorrected mass distribution data from 1-13C-glutamine. HCAECs were cultured with 0, 1, 2, or 5 mM of 1-^{13}C-glutamine for 24 hours, after which cells were collected, and the isotope distribution was determined using LC-MS. n=3 biological replicates.
